# Targeted Sonodynamic Therapy Platform for Holistic Integrative *Helicobacter pylori* Therapy

**DOI:** 10.1002/advs.202408583

**Published:** 2024-11-13

**Authors:** Xiaojing Yin, Yongkang Lai, Xinyuan Zhang, Tingling Zhang, Jing Tian, Yiqi Du, Zhaoshen Li, Jie Gao

**Affiliations:** ^1^ Department of Gastroenterology Shanghai Institute of Pancreatic Diseases Changhai Hospital Shanghai 200433 China; ^2^ Changhai Clinical Research Unit Changhai Hospital Naval Medical University Shanghai 200433 China; ^3^ Department of Gastroenterology Ganzhou People's Hospital Affiliated to Nanchang University Ganzhou 341000 China; ^4^ School of Health Science and Engineering University of Shanghai for Science and Technology Shanghai 200093 China; ^5^ National Key Laboratory of Immunity and Inflammation Naval Medical University Shanghai 200433 China; ^6^ Department of Pharmacy Shanghai Changhai Hospital the First Affiliated Hospital of Navy Medical University Shanghai 200433 China; ^7^ Shanghai Key Laboratory of Nautical Medicine and Translation of Drugs and Medical Devices Shanghai 200433 China

**Keywords:** autophagy, biofilms, *Helicobacter pylori* infection, holistic integrative medicine, sonodynamic therapy

## Abstract

*Helicobacter pylori* (*H. pylori*) is a primary pathogen associated with gastrointestinal diseases, including gastric cancer. The increase in resistance to antibiotics, along with the adverse effects caused by complicated medication protocols, has made the eradication of *H. pylori* a more formidable challenge, necessitating alternative therapeutics. Herein, a targeted nanoplatform is reported based on sonodynamic therapy, the chitosan‐conjugated fucose loaded with indocyanine green (ICG@FCS). It penetrates the gastric mucosa and homes in on *H. pylori* through dual targeting mechanisms: molecular via fucose and physical via ultrasound. Upon ultrasound activation, it generates singlet oxygen, effectively attacking planktonic bacteria, disrupting biofilms, and facilitating the clearance of intracellular bacteria by promoting autophagy, including multidrug‐resistant strains. The ICG@FCS nanoplatform minimally affects the gut microbiota and aids in gastric mucosa repair. a holistic integrative H. pylori therapy strategy is proposed that targets eradication while preserving gastrointestinal health. This strategy emphasizes the importance of maintaining patient health while eradicating the pathogen. This advancement is set to refine the comprehensive antibacterial approach, offering a promising horizon in the ongoing battle against antibiotic resistance and more effective gastric cancer prevention strategies.

## Graphical abstract

A molecularly targeted nanoplatform based on sonodynamic therapy offers an integrated treatment strategy that eradicates polymorphic *H. pylori*, repairs gastric mucosa, and preserves the gut microbiota, all while maintaining gastrointestinal health. This strategy presents a promising outlook for the ongoing fight against *H. pylori* antibiotic resistance and offers a more effective approach to gastric cancer prevention.

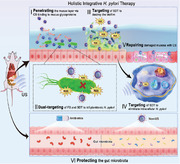

## 1 Introduction

*Helicobacter pylori (H. pylori)*, a spiral gram‐negative bacterium, is recognized as a high‐risk factor for gastric cancer. Globally, nearly 90% of noncardia gastric cancers are attributed annually to *H. pylori* infections.^[^ ^1^ ^]^ Our research team, led by Academician Zhaoshen Li from Shanghai Changhai Hospital in China, performed a large‐scale, nationwide, and house‐based epidemiology study of *H. pylori* infection and reported that the average individual infection rate of *H. pylori* in China is 40.66%, with a family average infection rate of 71.21%.^[^ ^2^ ^]^ In a 2023 review published in the journal *Gut*, Nobel laureate in Physiology or Medicine and *H. pylori* discoverer, Academician Marshall, advocated for our family‐based screening and the eradication of *H. pylori* as a viable and commendable strategy that merits widespread implementation.^[^ ^3^ ^]^ Extensive evidence supports the notion that eradicating *H. pylori* can notably decrease the incidence of gastric cancer, underscoring its critical role in cancer prevention strategies.^[^ ^2^, ^4^ ^]^

In recent years, the concept of holistic integrative medicine (HIM) has emerged as a powerful correction to the traditional single‐modality treatment approach. HIM emphasizes a holistic view of the patient, advocating for the integration of multidisciplinary knowledge to optimize therapeutic outcomes.^[^ ^5^ ^]^ In our previous research, we highlighted the importance of HIM in various medical contexts. For example, we reviewed postoperative integrative management strategies for bone tumor therapy and bone regeneration, where innovative biomaterials not only target tumor cells but also promote bone regeneration and the repair of damaged tissues.^[^ ^6^ ^]^ Another example is the application of multifunctional hydrogels in melanoma treatment, which combines tumor eradication with tissue repair.^[^ ^7^ ^]^ Similarly, in the context of *H. pylori* infection treatment, while eradication is a priority, safeguarding the functionality of other patient organs is also paramount. Nonetheless, traditional antibiotic therapies face numerous limitations: 1) increasing antibiotic resistance;^[^ ^8^ ^]^ 2) difficulty penetrating *H. pylori* biofilms;^[^ ^9^ ^]^ 3) the ability of the bacterium to evade clearance by suppressing autophagy and secluding itself within cells;^[^ ^10^ ^]^ and 4) the substantial medication burden coupled with severe side effects.^[^ ^2c^ ^]^ Notably, antibiotic‐induced dysbiosis of the gut microbiota can inflict considerable harm on intestinal health during *H. pylori* eradication.^[^ ^11^ ^]^ This approach, which does not mitigate organ damage during bacterial infection treatment, falls short of achieving optimal cancer prevention outcomes.^[^ ^5a^ ^]^ Consequently, incorporating the principles of HIM into *H. pylori* treatment protocols, which has profound implications for both the management of *H. pylori* infections and broader strategies for gastric cancer prevention, is imperative.

Biomaterials, characterized by their ability to shield drugs from gastric acid, target lesions, modulate drug delivery, disrupt biofilms, exhibit unique antimicrobial properties, demonstrate excellent biocompatibility, and have emerged as promising agents for the eradication of *H. pylori*, particularly drug‐resistant strains.^[^ ^12^ ^]^ In our 2022 review, we underscored the potential of these biomaterials as viable alternatives to antibiotics, offering solutions to combat antibiotic‐resistant *H. pylori* strains while addressing intestinal disorders.^[^ ^13^ ^]^ Consequently, our team previously developed a matrix metalloproteinase‐responsive hydrogel based on ascorbyl palmitate (AP), which has been confirmed to possess the ideal therapeutic effect of completely eradicating *H. pylori* and protecting the gut microbiota.^[^ ^14^ ^]^ However, owing to the complexity of its components and the high cost of production, its clinical translation has been limited. Thus, our team is dedicated to the rigorous refinement of antimicrobial substances to develop novel nanomaterials with simplified components and high clinical translational potential. This nanomaterial integrates the HIM principles of precision‐targeted antimicrobial and organ protection, aiming to solve the problem of *H. pylori* drug resistance while achieving gastrointestinal organ protection and providing a safe and effective integrated treatment strategy for patients.

Sonodynamic therapy (SDT) is an emerging antimicrobial strategy that offers advantages such as noninvasiveness, deep tissue penetration, and targeted selectivity.^[^ ^15^ ^]^ It operates by activating a sonosensitizer with low‐intensity ultrasound (US), which induces energy transfer to molecular oxygen within the tissue, converting it from its ground state to highly reactive oxygen species (ROS), including singlet oxygen (^1^O_2_).^[^ ^16^ ^] 1^O_2_ plays a pivotal role in disrupting both bacteria and biofilms.^[^ ^17^ ^]^ It induces oxidative stress by damaging bacterial cell walls and membranes, leading to bacterial lysis. Additionally, ^1^O_2_ degrades the extracellular polysaccharide matrix, which is critical for biofilm stability, increasing the susceptibility of bacteria to immune responses and antimicrobial agents.^[^ ^17^, ^18^ ^]^ For example, Yu et al. developed a pH‐responsive ROS nanogenerator that effectively eradicated various multidrug‐resistant (MDR) *H. pylori* strains and disrupted biofilms through the synergistic effects of SDT and chemodynamic therapy (CDT).^[^ ^17b^ ^]^ Beyond its antibacterial efficacy, Liu et al. demonstrated in their SDT‐based study that SDT not only had no adverse effects on the gut microbiota but also increased the abundance of the probiotic Lactobacillus.^[^ ^19^ ^]^ This level of safety is crucial for future clinical translation. While these studies have shown that SDT can effectively eradicate *H. pylori* through ROS generation, the mucus penetration and targeting capabilities of the materials used remain underexplored. Both characteristics are essential for improving the antimicrobial effect of SDT and protecting normal tissues. This is because the gastric mucus layer has a unique defense mechanism that traps and removes exogenous substances, including drugs.^[^ ^20^ ^]^ The mucus permeability of a material is key to its ability to function in the gastric environment. Additionally, materials lacking targeting properties may result in nonselective ROS attack on normal cells, which can diminish therapeutic efficacy and potentially induce side effects. Therefore, the development of materials with mucus penetration and high targeting properties is paramount for increasing the therapeutic effectiveness of SDT.

**Scheme 1:**
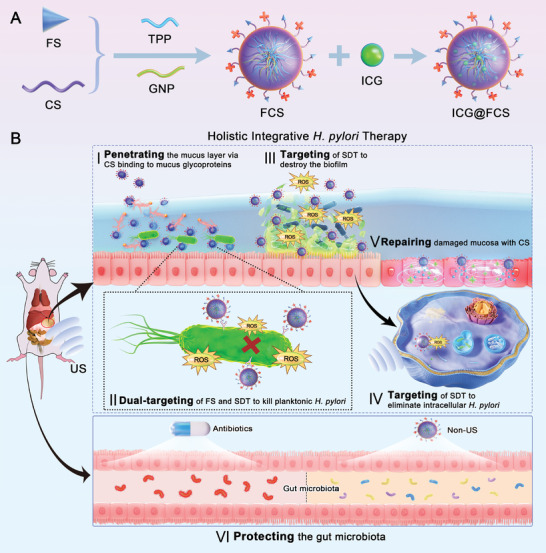
Illustrates the eradication of *H. pylori* via the SDT‐based ICG@FCS nanoplatform. (A) Schematic diagram of the preparation of FCS and ICG@FCS. (B) ICG@FCS penetrates the mucus layer, specifically recognizes and targets *H. pylori*, generates ROS under US stimulation to exert antimicrobial activity, eradicates biofilms, promotes autophagy to clear intracellular bacteria, and simultaneously repairs the gastric mucosa and protects the gut microbiota.

Herein, this study represents the inaugural report of a novel sonodynamic nanoplatform, denoted as ICG@FCS, which exhibits four properties that are necessary for holistic therapy of *H. pylori*: remarkable efficacy in penetrating the gastric mucosa, precise targeting toward bacteria, ability to eradicate intracellular pathogens, and facilitate gastric mucosal repair. Collectively, these distinct features render ICG@FCS a novel holistic therapeutic strategy for the efficient eradication of *H. pylori*. The ICG@FCS nanoplatform is engineered with indocyanine green (ICG), a sonosensitizer known for its safety and FDA approval for clinical use.^[^ ^21^ ^]^ It is covalently bonded with chitosan (CS) and fucose (FS), two nontoxic polysaccharides. Owing to its cationic properties, CS effectively binds to anionic gastric mucus, enhancing penetration and adhesion to the mucosa.^[^ ^22^ ^]^ Moreover, FS specifically targets the lectin receptor on *H. pylori*, providing a molecular targeting mechanism.^[^ ^23^ ^]^ US activation of ICG@FCS triggers the generation of ^1^O_2_, which is instrumental in eliminating both planktonic bacteria and biofilms while also inducing autophagy to clear intracellular pathogens.^[^ ^24^ ^]^ This dual‐targeting approach ensures precise delivery to *H. pylori* colonies, while controlled SDT and the inherent safety of the materials protect gastrointestinal organs (**Scheme** **1**).

## 2 Results and Discussion

### 2.1 Synthesis and Characterization of ICG@FCS

**Figure 1:**
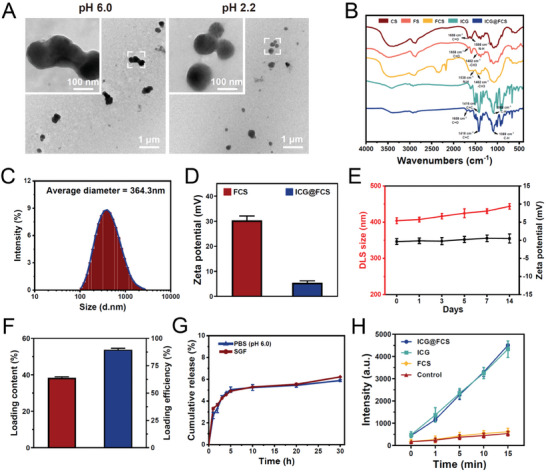
Characterization of the ICG@FCS nanoplatform. A) TEM images of ICG@FCS at pH 6.0 (left) and 2.2 (right). Main images: bars represent 1 µm; enlarged images: bars represent 100 nm. B) FTIR spectra (CS, FS, FCS, ICG, ICG@FCS). C) Particle size distribution of ICG@FCS. D) Zeta potentials of FCS and ICG@FCS. E) Stability assessments over a 14‐day period, including changes in the particle size (red line) and zeta potential (black line) of ICG@FCS. F) Drug loading content and loading efficiency of ICG in ICG@FCS. G) In vitro release rate of ICG in PBS (pH = 6.0) and SGF. H) ^1^O_2_ generation of H_2_O, FCS, ICG, and ICG@FCS.

The synthesis strategy of ICG@FCS is illustrated in Scheme 1. Briefly, the process involves the covalent conjugation of CS and FS, followed by sequential physical and chemical cross‐linking facilitated by tripolyphosphate (TPP) and genipin (GNP), forming a nanoshell (FCS) with superior stability and acid resistance. Next, ICG was encapsulated within the shell to obtain the final ICG@FCS nanoplatform. As shown in the transmission electron microscopy (TEM) images in **Figure** **1**A, at pH 6.0 and 2.2, ICG@FCS exhibited a relatively uniform spherical morphology without degradation. Fourier transform infrared (FTIR) spectroscopy revealed that the transmission peak at 1658 cm^−1^ corresponded to the stretching vibration of C═O in the amide I band of CS, whereas the peak at 1598 cm^−1^ was attributed to N‒H stretching in the amide II band. The FS spectra revealed bending vibrations of C═O and ─CH_3_ at ≈1658 and 1462 cm^−1^, respectively. In the spectra of FCS, the characteristic peak of ‐NH bending in CS shifted from 1598 to 1538 cm^−1^, and a peak corresponding to ─CH_3_ bending at 1462 cm^−1^ emerged in FS, indicating successful conjugation. Ultimately, the FTIR spectrum of ICG@FCS exhibited characteristic peaks for C═C at 1416 cm⁻¹ and C─H at 1088 cm⁻¹, aligning with the spectral features of ICG and confirming the successful encapsulation of ICG (Figure 1B). Figure 1C,D and Figure S1 (Supporting Information) show that the average particle sizes of FCS and ICG@FCS were 283.1 and 364.3 nm, respectively, with average potentials of +30.3 and +5.4 mV, respectively. Collectively, these findings confirmed the successful synthesis of the ICG@FCS nanoplatform.

Furthermore, we conducted preliminary assessments to validate the stability of the ICG@FCS particles when they were stored at room temperature under neutral conditions for 14 days, and the results indicated no significant alterations in particle size or zeta potential over time (Figure 1E). Additionally, the drug loading content and loading efficiency of ICG in ICG@FCS were 38.39% and 89.57%, respectively (Figure 1F). Given the focus of our research on mimicking the acidic gastric environment, we investigated the in vitro release profile of ICG from ICG@FCS under PBS (pH 6.0) and simulated gastric fluid (SGF) conditions (Figure 1G; Figure S2, Supporting Information). The findings revealed that the in vitro release rates of ICG in both media were consistently less than 5.5%, which is indicative of the nanoplatform's superior resistance to degradation by digestive enzymes and acidic conditions, which is conducive to effective drug delivery.

Following the characterization of the physical structure and chemical composition, as well as the assessment of stability, we quantified the production of ^1^O_2_ using a molecular probe, singlet oxygen sensor green (SOSG), for ICG, FCS, and ICG@FCS (Figure 1H). The results demonstrated that the encapsulation of ICG within the nanoshell did not compromise ^1^O_2_ generation, as ICG@FCS maintained a comparable level of ^1^O_2_ production to that of free ICG, which was directly proportional to the duration of US.

The highly acidic environment of the stomach poses a significant challenge for the delivery of orally administered drugs. Once drugs enter the stomach, they are rapidly eroded by gastric acid, leading to substantial degradation of their active ingredients and consequently reducing their bioavailability.^[^ ^20c^ ^]^ TPP and GNP are renowned for their excellent biocompatibility and are commonly utilized to increase the stability of materials and improve drug delivery efficiency. Research has indicated that when an acid‐treated CS droplet is added to a TPP cross‐linking agent solution, the free amine cations of CS are attracted electrostatically to the anions of TPP, which undergo physical cross‐linking to form tightly bound nanospheres.^[^ ^25^ ^]^ Furthermore, the primary amine groups of CS can undergo chemical cross‐linking reactions with GNP, which further strengthens the stability of the nanoshell, providing additional mechanical strength and resistance to dissociation.^[^ ^26^ ^]^ This dual‐cross‐linking mechanism ensures the stability and structural integrity of ICG@FCS under various environmental conditions. Moreover, compared with that of free ICG, the generation of ^1^O_2_ from the nanoplatform encapsulated within the nanoshell was not inhibited; it even tended to surpass that of free ICG over time. This phenomenon may be attributed to the fact that ICG is highly prone to inactivation at room temperature, but encapsulation within the nanoshell extends its circulation time within the body, thereby enhancing its drug utilization rate. Most notably, the materials we use are not only safe and cost‐effective but also scalable for mass production. These characteristics endow our material with substantial potential for clinical application.

### 2.2 Evaluation of Cytotoxicity, Mucosal Penetration, and Targeting Ability

**Figure 2:**
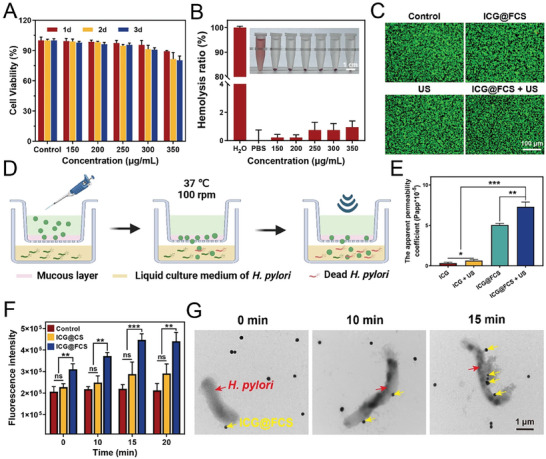
The cellular safety, mucus penetration, and targeting specificity were evaluated. A) Viability of HFE‐145 cells treated with different concentrations of ICG@FCS for 1, 2, and 3 days. B) Hemolysis rates of ICG@FCS at different concentrations (scale bar: 1 cm). C) Live/dead cell staining after coculture with ICG@FCS, US, or ICG@FCS+US for 3 days (scale bar: 100 µm). D) Schematic diagram of the mucus barrier in the in vitro transwell model. E) Papp values of ICG, ICG+US, ICG@FCS, and ICG@FCS+US across the mucus layer. F) Fluorescence intensity of ICG@CS and ICG@FCS coincubated with *H. pylori* for 0, 10, 15, or 20 min. G) TEM images of ICG@FCS co‐incubated with *H. pylori* for 0 (left), 10 (middle), or 15 min (right) (scale bar: 1 µm). All US treatments were performed for 10 min. The data are presented as the means ± SDs (*n* = 3), *p < 0.05, **p < 0.01, ***p < 0.001. ns, not significant.

Prior to investigating the sonodynamic effects of the material, we assessed its cytocompatibility, mucosal permeability, and targeting ability to ensure that the material could penetrate the mucous barrier and target *H. pylori* while not harming gastric epithelial cells. To accomplish this goal, HFE‐145 gastric epithelial cells were exposed to varying concentrations of ICG@FCS, and cell viability and survival rates were measured at 1, 2, and 3 days posttreatment via the CCK‐8 assay and live/dead cell staining. The results indicated that when the ICG@FCS concentration was 300 µg mL^−1^, the cell viability remained above 90% after 3 days (**Figure** **2**A; Figure S3, Supporting Information). Subsequent hemolysis assays also demonstrated the excellent blood compatibility of ICG@FCS at a concentration of 300 µg mL^−1^, with an average hemolysis rate of only 0.75% (Figure 2B). Furthermore, we preliminarily evaluated the cytotoxicity of ICG@FCS after different durations of US treatment. After the gastric epithelial cells were cocultured, they were subjected to US for 1, 5, 10, or 15 min (1 MHz, 1.5 W cm^−2^). A 10 min US treatment was the threshold for maintaining cell viability above 90% (Figure S4, Supporting Information). In conjunction with further exploration of the duration of US in subsequent antimicrobial experiments, the optimal conditions were identified as a 300 µg mL^−1^ material concentration combined with a 10 min US treatment. Additionally, after the cells were cocultured with ICG@FCS, US, or ICG@FCS+US (US treatment for 10 min) for 3 days, the proportion of surviving cells (green fluorescence) in all groups exceeded 90% (Figure 2C). These results collectively highlighted the excellent cytocompatibility of these materials. In pursuit of enhancing the efficacy against *H. pylori* by ensuring the penetration of the mucosal layer, this study evaluated the mucosal permeability of free ICG, ICG+US, ICG@FCS, and ICG@FCS+US (US treatment for 10 min) via the transwell system (Figure 2D). The apparent permeability coefficient (Papp) of ICG@FCS surpassed that of free ICG (*p *< 0.001), and SDT further increased the permeability (*p *< 0.01) (Figure 2E). Viability assays of *H. pylori* within the submucosa demonstrated that the application of ICG@FCS+US resulted in the most significant reduction in bacterial viability, highlighting the optimal combination of penetration and antimicrobial efficacy of this approach (Figure S5, Supporting Information).

Subsequently, we explored the targeting ability of ICG@FCS. *H. pylori* was incubated with PBS, ICG@CS (without FS), or ICG@FCS for 0, 10, 15, or 20 min, followed by centrifugation to remove unbound material, and the remaining fluorescence intensity of the bacterial suspension was measured. Quantitative analysis of the fluorescence intensity revealed no statistically significant difference between the ICG@CS group and the control group. In contrast, the fluorescence intensity of ICG@FCS, which contains FS, was significantly greater (*p* < 0.01). Additionally, there was no significant difference in fluorescence intensity between the 15 and 20 min incubation times, indicating that effective targeting could be achieved within 15 min (Figure 2F). We further visualized the interaction between ICG@FCS and *H. pylori* through TEM, which revealed that the highest degree of material adhesion to the bacteria occurred after 15 min of coincubation (Figure 2G). A comparison of TEM images between US exposure alone for 10 min and US applied after 15 min of material incubation revealed that US alone did not cause damage to the bacteria, whereas the combination of material and US led to more substances adhering to the bacterial surface, resulting in complete destruction of bacterial morphology (Figure S6, Supporting Information).

*H. pylori* is known to inhabit the niche between the gastric mucus layer and the gastric epithelium.^[^ ^27^ ^]^ Given the ability of the mucous layer to capture and clear exogenous substances, including drugs, penetrating this layer to achieve effective drug delivery is a critical challenge to overcome in the eradication of *H. pylori*. Khurshid et al. reviewed the excellent mucosal penetration and mucoadhesive properties of CS, highlighting the electrostatic interactions between the positively charged amino groups (‐NH) of CS and the negatively charged sialic acid residues of mucins, which effectively enhance the residence time and mucoadhesion of drugs in the gastrointestinal tract.^[^ ^22a^ ^]^ Our experiments also demonstrated that, compared with free ICG, ICG@FCS exhibited significantly better mucosal permeability. Furthermore, the permeability was enhanced when ICG@FCS was combined with US, likely because the mechanical vibrations of US promote the looseness of mucus, thereby accelerating the rate of drug penetration. After successfully penetrating the mucous layer, the targeted action of the drug on *H. pylori* becomes particularly important. This not only improves the efficiency of drug utilization but also reduces the risk of bacteria developing drug resistance. Research indicates that the surface of *H. pylori* contains lectins or adhesins that can specifically recognize and bind to carbohydrate receptors, including FS on the epithelial cells of the gastric mucosa.^[^ ^28^ ^]^ We also confirmed that materials loaded with FS could rapidly adhere to the surface of *H. pylori*, achieving molecular targeting. The physical targeting effect of combined SDT can significantly improve the efficiency of drug utilization, providing an effective therapeutic strategy for the eradication of *H. pylori*.

### 2.3 In Vitro Efficacy Against *H. pylori* in Diverse Forms

**Figure 3:**
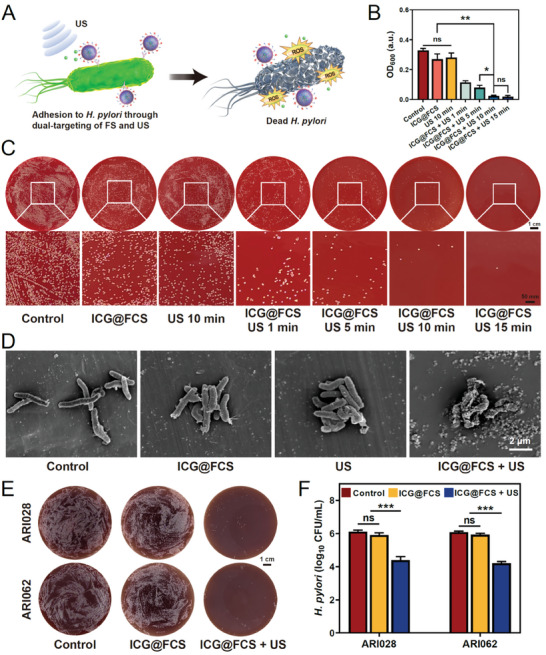
Antimicrobial efficacy of the nanoplatform in vitro. A) Schematic representation of in vitro antimicrobial activity. B) OD_600_ values of *H. pylori*. C) Colony plate images of *H. pylori* (scale bar: 1 cm). D) Morphological changes in *H. pylori* observed in SEM images of ICG@FCS, US 10 min, or ICG@FCS + US 10 min (scale bar: 2 µm). Colony plate images E) and colony counts F) of two drug‐resistant bacteria cocultured with ICG@FCS, US 10 min, or ICG@FCS + US 10 min. The data are presented as the means ± SDs (*n* = 3), *p < 0.05, **p < 0.01, ***p < 0.001. ns, not significant.

As illustrated in the bactericidal mechanism diagram we presented (**Figure** **3**A), ICG@FCS is capable of penetrating the mucus layer, adhering to the surface of *H. pylori*, and generating a substantial amount of ^1^O_2_ upon activation by extracorporeal US to eradicate the bacteria. To determine the optimal US exposure time, we measured the optical density (OD) at 600 nm of *H. pylori* after different treatment periods. The results indicated that neither ICG@FCS nor US alone had significant antimicrobial effects compared with those of the untreated control group. However, the bacterial concentration in the ICG@FCS+US treatment group was inversely proportional to the duration of US exposure, with the most potent antibacterial effects observed at 10 and 15 min, and there was no statistically significant difference between the two groups (Figure 3B). Colony counting and quantitative analysis on agar plates revealed similar results (Figure 3C; Figure S7, Supporting Information). Furthermore, live/dead bacterial staining further confirmed the excellent antibacterial activity of ICG@FCS+US at 10 min, characterized by reduced green fluorescence, indicating a significant decrease in viable *H. pylori* after treatment (Figure S8, Supporting Information). Therefore, for safety considerations, we selected 10 min as the final treatment duration. On the basis of these results, we consistently applied the 10 min US exposure in all subsequent experiments. Scanning electron microscopy (SEM) was subsequently employed to examine *H. pylori* treated with ICG@FCS, US, and ICG@FCS+US. Compared with those in the untreated control group, only minor morphological alterations, such as slight retraction of the bacterial body and curling or disappearance of flagella, were observed after treatment with ICG@FCS or US alone. However, the ICG@FCS+US group displayed pronounced membrane damage and cell lysis, accompanied by substantial leakage of the intracellular contents (Figure 3D). In addition to assessing the bactericidal effect against the standard strain of *H. pylori* (ATCC 43504), we extended our investigation to encompass two clinical drug‐resistant strains (ARI028 and ARI062). In contrast to the results of the control group, the application of ICG@FCS resulted in a modest decrease in bacterial count, which was not statistically significant. Conversely, following combination with SDT, there was a significant reduction in the counts of both strains (Figure 3E,F). These findings demonstrated that the combined therapy had an analogous bactericidal effect on drug‐resistant strains.

**Figure 4:**
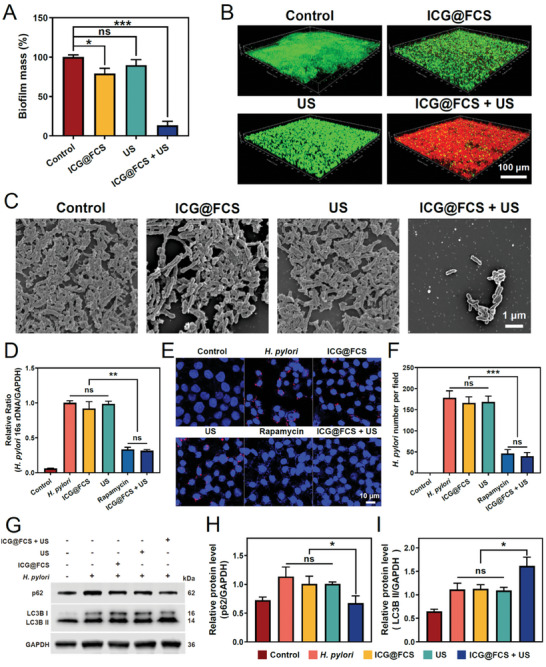
Antibiofilm and anti‐intracellular bacterial effects. A) Biofilm eradication detected by crystal violet staining. B) CLSM images of biofilms stained with SYTO 9 (green: live bacteria) and PI (red: dead bacteria) (scale bar: 100 µm). C) SEM images of biofilms (scale bar: 1 µm). D) Relative expression of intracellular *H. pylori* DNA. E) CLSM images of the intracellular *H. pylori* content in cells, showing cell nuclei (blue) and *H. pylori* (red) (scale bar: 10 µm). F) Whole‐cell lysates collected after coculture of an intracellular *H. pylori* model with nanoparticles and detection of LC3B and P62 protein levels by Western blotting; quantification of relative LC3B‐II (G) and P62 protein levels (H). All US treatments were performed for 10 min. The data are presented as the means ± SDs (*n* = 3), *p < 0.05, **p < 0.01, ***p < 0.001. ns, not significant.

*H. pylori* can form biofilms, which promote its survival and colonization, shielding it from external assaults and perpetuating a detrimental cycle.^[^ ^9^, ^29^ ^]^ Biofilm eradication is of paramount importance for the prevention of chronic and recurrent bacterial infections. We first quantitatively analyzed the inhibitory effect of the treatment group on biofilm formation through crystal violet staining experiments. Compared with the control, ICG@FCS moderately inhibited biofilm formation, reducing the membrane content by 20.82% (*p *< 0.05). The US group, influenced by mechanical effects, exhibited a 10.2% reduction, which was not statistically significant. In contrast, upon the integration of SDT, the destruction of the biofilm was much more extensive, leaving only 13.18% of the original content (*p *< 0.001) (**Figure** **4**A). Additionally, confocal laser scanning microscopy (CLSM) was used for live/dead staining of biofilms. The results showed that ICG@FCS or US alone could mildly disrupt biofilms, but the effects were limited, with considerable green fluorescence still observable. In comparison, the density and viability of biofilms were significantly diminished after treatment with ICG@FCS+US (Figure 4B). SEM observations of biofilm morphology revealed a trend similar to that of crystal violet staining and CLSM. Under the action of ICG@FCS combined with SDT, the biofilms of *H. pylori* were completely damaged and cleared (Figure 4C).

*H. pylori* is primarily an extracellular bacterium, yet increasing evidence suggests that it can also survive and colonize gastric epithelial cells and macrophages.^[^ ^30^ ^]^ After extracellular antibiotic treatment ends, bacteria can be released from the sanctuary of gastric epithelial cells and re‐enter the extracellular environment, initiating a new cycle of infection.^[^ ^30^, ^31^ ^]^ Therefore, eliminating intracellular *H. pylori* is crucial for reducing the risk of persistent infection and recurrence. In this study, we first utilized qPCR technology to detect the expression levels of *H. pylori* 16S rDNA within cells. The results indicated that the expression of 16S rDNA in *H. pylori*‐infected cells was significantly greater than that in the control group (*p* < 0.001). Compared with no treatment, treatment with ICG@FCS or US alone did not result in a significant change in the expression levels of 16S rDNA. However, when ICG@FCS was combined with US, the expression level of 16S rDNA decreased to a level similar to that in the rapamycin‐treated group, which promoted autophagy. This finding suggested that the platform may clear intracellular bacteria by promoting cellular autophagy (Figure 4D). Additionally, similar trends were observed through CLSM and quantitative analysis (Figure 4E,F). To further verify these findings, we conducted Western blot analysis. The results revealed that the p62 protein levels in *H. pylori*‐infected cells were significantly greater than those in uninfected control cells, as was the increase in the level of the autophagy‐related protein LC3B II. These results suggest that *H. pylori* may evade host clearance mechanisms by inhibiting the early autophagy process in host cells. Cells treated with either ICG@FCS or US alone showed trends similar to those of the *H. pylori*‐infected group, indicating that single treatment methods have limited efficacy in clearing intracellular bacteria. However, when ICG@FCS and US were combined, the protein expression level of p62 decreased significantly to levels close to those of the control group, whereas the expression of LC3B II further increased. This further confirmed the potential of the platform to promote cellular autophagy to clear intracellular bacteria (Figure 4G–I).

The data presented above corroborated the superior bactericidal efficacy of combination therapy involving ICG@FCS with SDT against *H. pylori* in diverse states. Notably, the bactericidal efficacy of ICG@FCS or US alone is quite limited. For US alone, the limited bactericidal effect may be attributed to the localized thermal effect it induces. As for CS‐based nanomaterials, which have been reported in previous studies to exhibit some degree of antibacterial activity against *H. pylori*,^[^ ^32^ ^]^ yet the limited antibacterial efficacy observed in our system can be attributed to several factors. First, in our study, CS was utilized as a component that binds with negatively charged TPP and FS, with a primary focus on enhancing the penetration of nanomaterials through the gastric mucus layer.^[^ ^33^ ^]^ Second, the concentration of CS in our formulation was very low, and it was not used as the main antimicrobial agent. This explains why our results showed that the antibacterial effect of the nanoparticles was limited in the absence of US. Therefore, the generation of ^1^O_2_ by US‐activated ICG is the core of our eradication strategy, providing a more focused and efficient method for bacterial elimination.

In addition, biofilms are primarily composed of an extracellular polysaccharide matrix, which is critical for maintaining biofilm structural stability.^[^ ^34^ ^]^ Although previous studies have demonstrated that ^1^O_2_ can degrade the polysaccharide matrix, thereby compromising biofilm integrity and rendering the enclosed bacteria more susceptible to antimicrobial agents, these studies often rely on chemical agents or photodynamic therapy (PDT) to generate ^1^O_2_.^[^ ^18^, ^35^ ^]^ For instance, Xiu et al. reported that PDT could eradicate methicillin‐resistant *Staphylococcus aureus* biofilms by generating ^1^O_2_.^[^ ^36^ ^]^ However, this method is limited in treating deep‐seated infections because of the inability of light to penetrate deeper tissues. Additionally, while some novel nanomaterials, such as silver nanoparticles, have shown potential in disrupting biofilms, their associated toxicity cannot be overlooked.^[^ ^37^ ^]^ In contrast, the ICG@FCS nanoplatform utilizes controllable SDT to achieve efficient *H. pylori* biofilm eradication, demonstrating significant safety advantages.

Ultimately, autophagy serves as a cellular mechanism for self‐repair and self‐cleansing.^[^ ^10^ ^]^ Studies have indicated that *H. pylori* can persist within cells by suppressing autophagy and accumulating p62, which can damage cellular DNA.^[^ ^38^ ^]^ Enhancing autophagy has been shown to be lethal to *H. pylori*.^[^ ^39^ ^]^ In our study, we observed a significant reduction in the number of intracellular bacteria following treatment with ^1^O_2_, an effect comparable to that of rapamycin, a well‐documented autophagy promoter that effectively eliminates intracellular bacteria. We hypothesize that the primary mechanism of intracellular bacterial eradication by the nanoparticles is through the promotion of autophagy facilitated by US‐induced ^1^O_2_ production, thereby diminishing *H. pylori* within cells. In summary, combined therapy targets the aggregation areas of *H. pylori* with precision, significantly amplifying the bactericidal effect against various forms of *H. pylori* by generating ^1^O_2_ through a dual‐targeting mechanism.

### 2.4 In Vivo Anti‐*H. pylori* Efficacy of ICG@FCS Combined with SDT

**Figure 5:**
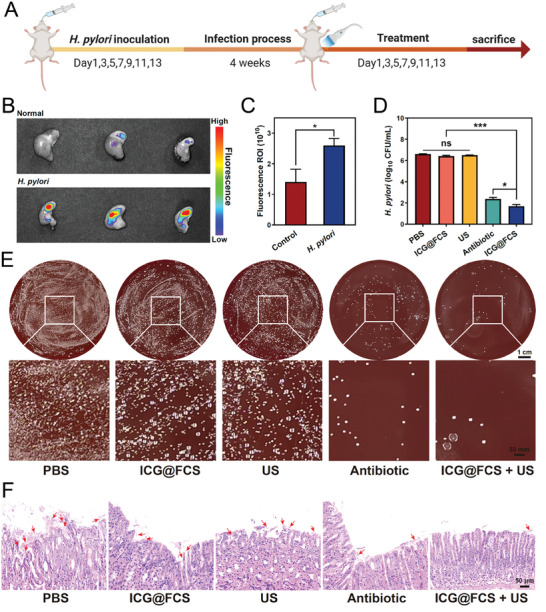
Anti*‐H. pylori* efficacy of the nanoplatform in vivo. A) Schematic diagram illustrating the construction and treatment of *H. pylori*‐infected mouse models. Imaging of gastric tissues via the IVIS imaging system B) and quantification of their fluorescence intensity C). Colony counts of *H. pylori*‐infected mouse models D) and plate images E) (scale bar: 1 cm, magnification scale bar: 50 mm). F) H&E staining of mouse gastric tissues (red arrows indicate *H. pylori*) (scale bar: 50 µm). All US treatments were performed for 10 min. The data are presented as the means ± SDs (*n* = 3), *p < 0.05, **p < 0.01, ***p < 0.001. ns, not significant.

Before evaluating in vivo antimicrobial efficacy, it is imperative to assess the toxicity of the drug. For this purpose, we established an *H. pylori* infection model in C57BL/6 mice. Four weeks after colonization in mice, *H. pylori* was successfully isolated and cultured on blood agar plates, with colonies appearing small and translucent and exhibiting slow growth. The cultured bacterial suspension was subsequently inoculated into a rapid urease test reagent, resulting in a color change of the indicator from yellow to purple‒red. Finally, histological sections of the gastric tissue from the mice were subjected to H&E staining, revealing that the bacteria were curved or spiral in shape and adhered to the gastric mucosa, accompanied by extensive inflammatory cell infiltration. Therefore, these three methods effectively confirmed the successful establishment of the *H. pylori* infection model in mice (Figure S9, Supporting Information). Then, the mice were randomized into five treatment groups, namely, the PBS, ICG@FCS, US, antibiotic, and ICG@FCS+US groups, with treatments given every other day for two weeks. The mice were euthanized on the second day after the completion of treatment (**Figure** **5**A). Histological examination of the hearts, livers, spleens, lungs, and kidneys of the mice revealed no pathological signs in the treatment groups, indicating that the ICG@FCS combined with SDT did not cause pathological damage to the major organs (Figure S10, Supporting Information). Additionally, physiological parameters such as blood biochemistry and body weight changes did not differ between the treatment groups and the control group (Figures S11 and S12, Supporting Information). These results demonstrated that ICG@FCS with SDT exhibited favorable biocompatibility in vivo.

Next, we evaluated the capacity of ICG@FCS combined with SDT to eradicate *H. pylori* in vivo. Using IVIS imaging, we observed robust fluorescence signals in the gastric tissues of *H. pylori*‐infected mice, which contrasted with the significantly diminished signals in uninfected controls; a statistically significant difference in the average signal intensity was observed (*p* < 0.05) (Figure 5B,C). These findings validated the ability of ICG@FCS to target the stomach, potentially enhancing the gastric retention time and therapeutic efficacy. Subsequently, the antimicrobial effects were further confirmed through plate culture and colony enumeration (Figure 5D,E). The *H. pylori* infection in the ICG@FCS+US group was significantly reduced compared to the PBS group (*p* < 0.001), whereas the treatment effects in both the ICG@FCS and US groups did not reach statistical significance. Furthermore, the reduction in CFU counts in the ICG@FCS+US group was more pronounced than that in the antibiotic group (*p* < 0.05). In addition, H&E staining to evaluate the condition of the gastric mucosa showed that in *H. pylori*‐infected mice treated with PBS, the gastric mucosa exhibited necrosis, extensive infiltration of inflammatory cells, and dense *H. pylori* colonization near the gastric mucosa and small pits (red arrows). In contrast, mice treated with US showed no significant changes, inflammation, and *H. pylori* were slightly decreased in the ICG@FCS group, and *H. pylori* colonization in the gastric mucosal epithelium was still observed in the antibiotic group. Notably, the mice treated with ICG@FCS+US displayed minimal inflammation and no evident *H. pylori* colonization (Figure 5F). Taken together, these results indicated that ICG@FCS combined with SDT had the strongest effect on *H. pylori* clearance and were effective in relieving gastric inflammation in vivo.

### 2.5 Evaluation of Gastric Mucosal Repair Capacity

**Figure 6:**
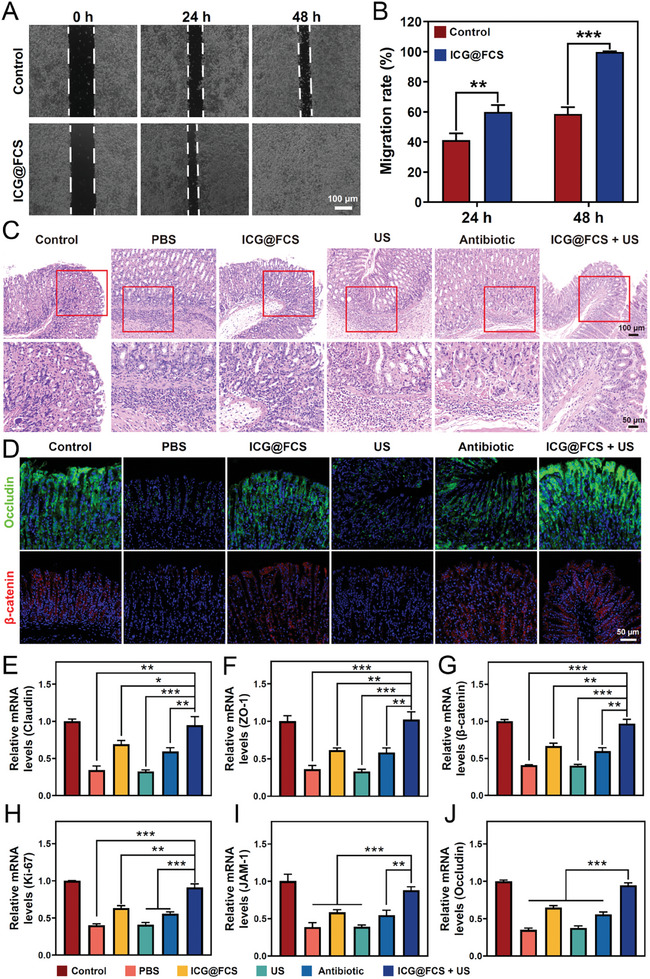
Effects of the nanoplatform on gastric mucosal repair. A) Images depicting cell migration (scale bar: 100 µm) and B) the corresponding quantification analysis after treatment with ICG@FCS. C) H&E staining of gastric tissues from mice (scale bar: 100 µm, magnification scale bar: 50 µm). D) Observation of epithelial repair in the mouse gastric mucosa via occludin fluorescence staining. Fluorescence staining of β‐catenin was performed to detect apoptosis of gastric epithelial cells (scale bar: 50 µm). Relative mRNA levels of Claudin E), ZO‐1 F), β‐catenin G), Ki‐67 H), JAM‐1 I), and Occludin J). All US treatments were performed for 10 min. The data are presented as the means ± SDs (*n* = 3), *p < 0.05, **p < 0.01, ***p < 0.001. ns, not significant.

*H. pylori*‐induced gastric mucosal damage can lead to chronic gastritis, peptic ulcers, and even gastric cancer. Therefore, the eradication of *H. pylori* while promoting gastric mucosal repair holds significant clinical significance in the treatment of gastric‐related diseases. This section describes the analysis of the mucosal repair ability of ICG@FCS combined with SDT. As shown in **Figure** **6**A,B, ICG@FCS promoted the migration of gastric mucosal epithelial cells, suggesting that these nanoparticles had the ability to promote mucosal repair. To further investigate the gastric mucosal healing status post‐*H. pylori* eradication, we conducted H&E staining and graded gastritis in accordance with the Sydney System. These findings revealed that while the PBS group displayed diffuse inflammatory cell infiltration in both the gastric mucosal and submucosal layers, the ICG@FCS and US groups presented localized inflammatory cell infiltration even after drug treatment. However, in the ICG@FCS+US group, the number of inflammatory cells in the gastric mucosa was significantly lower, the mucosa was intact, and the degree of infection was lower than that in the antibiotic group (Figure 6C). The gastritis score was also not different from that of the uninfected group (Figure S13, Supporting Information). Additionally, immunofluorescence staining was used to detect the expression levels of the intercellular adhesion proteins β‐catenin, Ki‐67, ZO‐1, and Occludin in gastric mucosal epithelial cells (Figure 6D; Figure S14, Supporting Information). When gastric epithelial cells were infected with *H. pylori*, their expression levels decreased due to the disruption of intercellular adhesion by *H. pylori*. However, after treatment with ICG@FCS, the antibiotic, or ICG@FCS+US, the expression of the cell proteins significantly increased, with the ICG@FCS+US group exhibiting the highest expression. Moreover, the qPCR results revealed the mRNA expression levels of proteins such as claudin and JAM‐1, revealing that the mRNA levels of these proteins were congruent with their respective protein expression profiles (Figure 6E–J; and Table S1, Supporting Information). After treatment with ICG@FCS+US, the gene expression levels tended to resemble those of normal gastric mucosal epithelial cells, and the differences between this group and the other treatment groups were statistically significant (*p* < 0.05). These results indicated that ICG@FCS combined with SDT exhibited the most effective gastric mucosal repair.

Gastric mucosal repair is an overlooked yet critical clinical issue. When *H. pylori* colonizes the surface of gastric epithelial cells, it releases toxins that damage these cells, leading to gastritis or peptic ulcers.^[^ ^40^ ^]^ Although eradicating *H. pylori* can reduce gastric mucosal damage, full mucosal recovery is not straightforward. Conventional treatment modalities, including mucosal protective agents and proton pump inhibitors (PPIs), are often limited by patient noncompliance and adverse effects.^[^ ^2c^ ^]^ The ability of CS, a naturally occurring alkaline polysaccharide, to neutralize gastric acid and suppress the secretion of gastric acid and pepsin, thereby facilitating gastric mucosal repair, has been validated by multiple studies.^[^ ^41^ ^]^ Furthermore, CS promotes the proliferation and migration of gastric mucosal epithelial cells, hastening the healing of damaged mucosa.^[^ ^42^ ^]^ In our investigation, the synergistic application of ICG@FCS with SDT markedly augmented the restoration and regeneration of the gastric mucosal epithelium. This enhanced effect may stem from two aspects: first, ICG@FCS, which serves as an antimicrobial nanoplatform, eradicates *H. pylori*, thereby mitigating gastric mucosal aggression and allowing the mucosal barrier to recover; second, CS augments mucosal repair through the stimulation of cellular proliferation and offers protective effects. This holistic integrative *H. pylori* therapy strategy, which combines bactericidal action with mucosal repair effects, not only effectively mitigates the symptoms of patients after *H. pylori* eradication but also reduces the reliance on additional mucosal reparative agents, underscoring its substantial clinical significance.

### 2.6 Effects on the Gut Microbiota and Transcriptome Analysis

**Figure 7:**
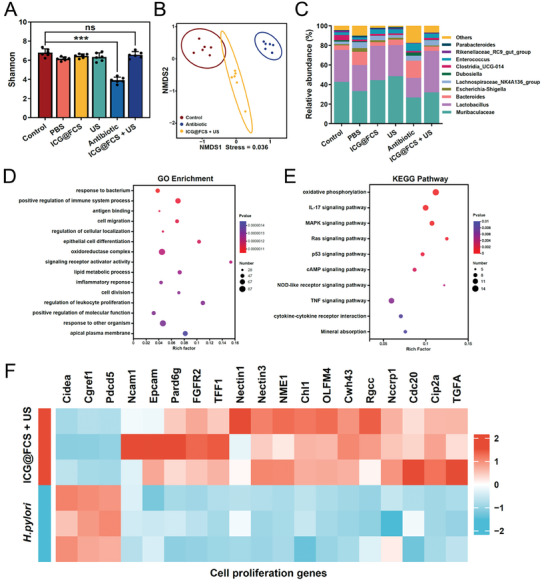
Effects on the gut microbiota and gastric transcriptome analysis. A) Determination of the Shannon index in mouse feces posttreatments to analyze microbial richness. B) NMDS analysis of intestinal microbiota β diversity after antibiotic and ICG@FCS+US treatments. C) Relative abundance of microbial communities in mouse feces after treatment determined by 16S rRNA sequencing. D) Bubble plot displaying the GO pathway enrichment analysis between the control and ICG@FCS+US group. E) Bubble plot displaying the KEGG pathway enrichment analysis between the control and ICG@FCS+US group. F) Gene expression analysis of cell proliferation regulation in the ICG@FCS+US and *H. pylori* (PBS) group of mice. All US treatments were performed for 10 min. The data are presented as the means ± SDs (*n* = 3), *p < 0.05, **p < 0.01, ***p < 0.001. ns, not significant.

After *H. pylori eradication*, antibiotic therapy may induce dysbiosis of the intestinal microbiota, potentially resulting in gastrointestinal adverse effects. Consequently, the impacts of posteradication on the intestinal microbiota are of paramount concern. To assess the effects of treatment on the intestinal microbiota, fecal samples were collected from mice within 48 hours posttreatment for 16S rRNA gene sequencing. This analysis aimed to evaluate the abundance, diversity, and community structure of intestinal bacteria across different treatment groups (Figure S15, Supporting Information). Analysis of the α diversity of the intestinal flora (Chao 1, Shannon, and Simpson indices) indicated that the α diversity of the intestinal flora in the ICG@FCS+US group did not significantly differ from that in the healthy group, whereas the antibiotic group presented markedly reduced α diversity (**Figure** **7**A; Figure S16, Supporting Information). Principal coordinate analysis (PCoA) and nonmetric multidimensional scaling analysis (NMDS) of the β diversity of the gut microbiota based on unweighted UniFrac distance metric also revealed that the β diversity of the ICG@FCS+US group was more similar to that of the healthy group than to the antibiotic group. Pairwise PERMANOVA revealed a *p value* of 0.103 between the ICG@FCS+US group and the control group and a *p value* of 0.001 between the antibiotic group and the control group (Figure 7B; Figure S17, Supporting Information). These findings further support the notion that, compared with antibiotic treatment, ICG@FCS+US treatment has a minimal effect on the gut microbiota of mice. Overall, these findings suggested that the interference of ICG@FCS combined with SDT on the diversity of the gut microbiota was significantly reduced. Furthermore, we quantified the relative abundance of the intestinal flora. At the genus level, compared with the control group, the antibiotic group displayed a notable increase in the proportion of the pathogenic bacteria Enterococcus and Bacteroides, along with a significant decrease in the proportions of the beneficial bacteria Lactobacillus and Muribaculaceae. In contrast, the differences in the ICG@FCS+US group were minimal, and no occurrence or increase in pathogenic bacteria was observed (Figure 7C). These findings further support the notion that ICG@FCS combined with SDT have no adverse effects on the community structure of the gut microbiota.

The use of antimicrobial agents is a significant cause of gut microbiota dysbiosis and is also one of the contentious issues regarding whether *H. pylori* should be eradicated universally in the population.^[^ ^11^ ^]^ Studies have shown that the eradication of *H. pylori* can damage healthy gut microbiota, with particularly evident short‐term effects, and some patients may even remain in an “irritable” state of the gut microbiota 4 years after treatment.^[^ ^43^ ^]^ However, in vivo research has indicated that the combination of ICG@FCS with SDT is capable of eradicating *H. pylori* with negligible disturbance to the intestinal environment. This positive outcome can likely be attributed to the unique design and application of our nanoplatform. This approach integrates the molecular targeting effect of FS with the physical targeting effect of SDT, ensuring that the therapeutic process precisely targets the stomach, the specific area where *H. pylori* aggregates. Moreover, the high safety profile of the material and the controllable process of SDT significantly reduce potential adverse effects on gastrointestinal organs. These advantages demonstrate the potential of holistic integrative *H. pylori* therapy in the field of *H. pylori* treatment. In addition, we further analyzed the antimicrobial mechanism of ICG@FCS combined with SDT via transcriptome sequencing. Enriched Gene Ontology (GO) analysis was performed on differentially expressed genes (DEGs) obtained from the control versus ICG@FCS+US genomes to elucidate the relevant functional pathways in which DEGs may be involved in gene regulation. As shown in Figure 7D, GO analysis revealed pathways related to the response to bacteria, positive regulation of immune system processes, cell migration, epithelial cell differentiation, and regulation of leukocyte proliferation. These findings are consistent with the antimicrobial and reparative functions that we previously established. Additionally, the Kyoto Encyclopedia of Genes and Genomes (KEGG) pathway enrichment analysis revealed that the ICG@FCS+US genome is involved mainly in cell proliferation‐related signaling pathways (the MAPK signaling pathway and Ras signaling pathway) and inflammation‐related pathways (the TNF signaling pathway and the IL‐17 signaling pathway) (Figure 7E), suggesting that our nanoplatform has anti‐inflammatory and reparative functions. We subsequently conducted GO analysis on DEGs related to cell proliferation between H. pylori and the ICG@FCS+US genome. As shown in Figure 7F and *H. pylori* infection significantly upregulated the expression of genes that promote cell apoptosis (Cidea, Cgref1, and Pdcd5) but downregulated the expression of genes that promote cell proliferation (Pard6 g, FGFR2, TFF1, etc.). Treatment with ICG@FCS+US resulted in the downregulation of these proapoptotic genes and the upregulation of genes promoting proliferation. This finding suggested that ICG@FCS+US had a reparative effect on the gastric mucosa.

On the basis of these findings, we posit that the concurrent administration of ICG@FCS and SDT has an antimicrobial effect while fostering mucosal restoration through the upregulation of genes related to cell proliferation. This holistic integrative therapeutic strategy for *H. pylori* infection not only effectively mitigates patient symptoms subsequent to *H. pylori* eradication but also diminishes the need for additional mucosal reparative pharmaceuticals. It encapsulates the holistic integrative medical concept that encompasses both antimicrobial action and organ preservation, thereby demonstrating its substantial clinical importance.

## 3 Conclusion

Eradicating *H. pylori* is of paramount importance for the prevention and treatment of various gastric diseases, including gastric cancer. In this study, we developed a safe antimicrobial nanomaterial with simple and safe components that could show high potential for clinical translation. This study pioneers the integration of gastric mucosal penetration, dual targeting of bacteria and biofilms at both the physical and molecular levels, intracellular pathogen autophagy, and gastric mucosal repair, offering a comprehensive approach to *H. pylori* treatment. Our research demonstrated that the SDT‐based ICG@FCS nanoplatform exhibited excellent antimicrobial effects both in vitro and in vivo, effectively targeting various forms of *H. pylori*. Through the synergistic effects of CS and SDT, ICG@FCS can penetrate the gastric mucus layer and, with the dual targeting action of FS and SDT, can precisely locate the surface of *H. pylori*. Under US stimulation, ICG produces ^1^O_2_, effectively attacking planktonic bacteria, disrupting biofilms, and facilitating the clearance of intracellular bacteria through the promotion of autophagy. Additionally, SDT achieves site‐specific cytotoxicity by generating ^1^O_2_ in response to a sonosensitizer without inducing bacterial resistance.^[^ ^44^ ^]^ Given the controllable activation of SDT combined with the targeting and safety of the material, this strategy has a negligible impact on the abundance, function, and species diversity of the gut microbiota and can promote complete healing of the gastric mucosa. Based on the above findings, for the first time, we introduce the principles of HIM for the treatment of *H. pylori* and propose a novel treatment strategy — holistic integrative *H. pylori* therapy. This strategy emphasizes the importance of maintaining overall patient health while eradicating the pathogen.

It is crucial to acknowledge the limitations of this study. First, there are inherent limitations in translating findings from preclinical animal models to human therapy. Specifically, significant anatomical and physiological differences exist between the stomachs of mice and humans, which may influence therapeutic outcomes. For example, variations in gastric pH, immune responses, and microbiota composition between species could alter the interactions of the nanoplatform with *H. pylori* and its biofilms. Additionally, scaling up the production of the nanoplatform while ensuring consistency in its performance and efficacy remains a significant challenge. Finally, SDT requires specialized US equipment and precise control over US parameters, which may limit the widespread application of this method in clinical settings. Therefore, further research is needed to optimize these technical aspects and ensure the safe and effective application of this therapy in humans.

Beyond its efficacy against *H. pylori*, our platform exhibits substantial promise in managing a spectrum of bacterial infections. Emerging research has strongly correlated the presence of *Streptococcus anginosus* with the etiology of gastric cancer, underscoring its potential contribution to disease initiation and progression.^[^ ^45^ ^]^ In light of these insights, we hypothesize that the antimicrobial properties of the ICG@FCS nanoplatform can be fine‐tuned to selectively engage and neutralize *Streptococcus anginosus*. Furthermore, we posit that the platform's utility may be broadened to encompass other pathogens implicated in gastrointestinal disorders by modulating or enhancing the structural elements pivotal to target specificity. This innovative strategy, when integrated with our comprehensive antimicrobial approach, transcends traditional antimicrobial therapies and represents a paradigm shift, potentially paving the way for significant advancements in the eradication of pathogenic bacteria and the prevention of gastric cancer.

## Conflict of Interest

The authors declare no conflict of interest.

## Supporting information



Supporting Information

## Data Availability

The data that support the findings of this study are available from the corresponding author upon reasonable request.
