# Supplementary material for: Targeted Sonodynamic Therapy Platform for Holistic Integrative Helicobacter pylori Therapy
Source: Adv Sci (Weinh). 2024 Nov 13;12(2):2408583. doi: 10.1002/advs.202408583 (PMC11727135; doi:10.1002/advs.202408583)
Supplement: Supplementary file 1 — Supporting Information [file ADVS-12-2408583-s001.docx]

**Supporting Information**

**Targeted Sonodynamic Therapy Platform for Holistic Integrative *Helicobacter pylori* Therapy**

Xiaojing Yin ^1^*, Yongkang Lai ^1,3^*, Xinyuan Zhang ^5^*, Tingling Zhang ^2,4^, Jing Tian ^7^, Yiqi Du ^1,2,4^†, Zhaoshen Li ^1,2,4^†, Jie Gao ^2,4,6^†

^1^ Department of Gastroenterology, Shanghai Institute of Pancreatic Diseases, Changhai Hospital, Shanghai, 200433, China

^2^ Changhai Clinical Research Unit, Changhai Hospital, Naval Medical University, Shanghai, 200433, China

^3^ Department of Gastroenterology, Ganzhou People’s Hospital Affiliated to Nanchang University, Ganzhou, 341000, China

^4^ National Key Laboratory of Immunity and Inflammation, Naval Medical University, Shanghai, 200433, China

^5^ School of Health Science and Engineering, University of Shanghai for Science and Technology, Shanghai, 200093, China

^6^ Shanghai Key Laboratory of Nautical Medicine and Translation of Drugs and Medical Devices, Shanghai, 200433, China.

^7^ Department of Pharmacy, Shanghai Changhai Hospital, the First Affiliated Hospital of Navy Medical University, Shanghai, 200433, China

* Xiaojing Yin, Yongkang Lai and Xinyuan Zhang contributed equally to this paper

†Correspondence to:

Prof Jie Gao, Changhai Clinical Research Unit, Shanghai Changhai Hospital, Naval Medical University, Shanghai 200433, China; gaojiehighclea@smmu.edu.cn; Telephone: 021-31166666; Fax: 021-31162332;

Prof Zhaoshen Li, Department of Gastroenterology, Changhai Hospital, Naval Medical University; 168 Changhai Road, Yangpu District, Shanghai 200433, China; chzshenli@163.com; Telephone: 021-31166666; Fax: 021-31162332;

Prof Yiqi Du, Department of Gastroenterology, Changhai Hospital, Naval Medical University, Shanghai 200433, China, Email: duyiqi@hotmail.com. Telephone: 021-31166666; Fax: 021-31162332.

**Experimental Section**

***Materials:*** L-Fucose (L809666) and tripolyphosphate (S817361) were purchased from Macklin Bio-Tech Co., Ltd. (Shanghai, China). Chitosan (deacetylation degree≥95%, viscosity 100-200 mPa s, C105799) and gentamicin (G1914) were acquired from Aladdin Bio-Chem Technology Co., Ltd. (Shanghai, China). Indocyanine green (BD134593), genipin (BD131935), and sodium cyanoborohydride (BD151416) were obtained from Bide-Pharm Technology Co., Ltd. (Shanghai, China). Clarithromycin (C9490), vancomycin (V8050), polymyxin B sulfate (P8350), trimethoprim lactate (T9170), and amphotericin B (A8251) were purchased from Sigma‒Aldrich (Shanghai, China). A Live/Dead Cell Staining Kit (40747ES76), Cell Counting Kit-8 (40203ES) and Hieff UNICON^®^ Universal Blue qPCR SYBR Green Master Mix (11184ES08) were purchased from Yeasen Biotech (Shanghai, China) Co., Ltd. The SYTO 9/PI Live/Dead Bacterial Double Stain Kit (MX4234-40T) was obtained from Moukang Biotech Co. (Shanghai, China). A rapid urease test kit was obtained from Bomeda Biotech Co. (Shandong, China). A BCA protein assay kit (23227) and TRIzol^TM^ (15596026) were obtained from Thermo Fisher Scientific, Inc. (Massachusetts, USA). All antibodies were purchased from Abcam. *H. pylori* strains (43504, TN2GF4, and PMSS1) and gastric epithelial cells (HFE-145) were obtained from the Institute of Digestive Diseases, the First Affiliated Hospital of Nanchang University. Clinical drug-resistant strains (ARI028 and ARI062) were obtained from the Department of Trauma Orthopedics, Shanghai Ruijin Hospital.

***Synthesis of ICG@FCS:*** Chitosan (CS) and fucose (FS) were conjugated via an N-alkylation reduction method.^[1]^ Briefly, 0.5 g of CS was dissolved in 50 mL of 1.0% dilute acetic acid. Subsequently, 10 mL of deionized water containing 0.5 g of FS and 1.0 g of sodium cyanoborohydride was added. The mixture was stirred continuously at room temperature for 6 hours to ensure that the reaction was complete. To eliminate unconjugated components, the sample was subjected to dialysis against 5 L of deionized water in the dark for one day, with water replacement every 2 hours for a total of five changes. Next, under constant magnetic stirring (600 rpm), tripolyphosphate (TPP) solution (0.25 mg/mL, 4 mL) was slowly added dropwise to the mixture at a rate of approximately 3 mL/min. Thereafter, a genipin (GNP) aqueous solution (0.5 mg/mL, 4 mL) was gradually introduced into the mixture, resulting in the formation of the FCS nanoshell. Finally, an ICG solution (5 mg/mL, 2 mL) was added to 4 mL of the aforementioned mixture and stirred at room temperature for 24 hours. The nanoparticles were further purified via centrifugation at room temperature via a 10 kDa centrifuge tube to remove excess free ICG.

***Characterization:*** Transmission electron microscopy (TEM) was used to examine the morphological characteristics of the nanoplatform via an FEI Talos F200X G2 microscope (USA, 200 kV). Structural analysis of the nanoplatform was performed through Fourier transform infrared (FTIR) spectroscopy performed on a Thermo Fisher Scientific Nicolet iS20 spectrometer (USA). Additionally, the diameter and zeta potential of the materials were characterized via a ZetaSizer Nano-ZS 90 instrument (Malvern Instruments, Malvern, UK).

The drug loading content and efficiency were determined following established methods from the literature.^[2]^ Briefly, 2 mL of various concentrations of ICG solutions (0.75, 1.5, 3, 4.5, 6, and 7.5 μg/mL in methanol) were added to the ICG@FCS solution. The mixture was stirred continuously under identical conditions for 24 hours. After centrifugation at 12,000 rpm for 15 minutes, the supernatant was collected. The absorbance of the supernatant at a wavelength of 781 nm was recorded via an ultraviolet‒visible‒near-infrared (UV‒Vis-NIR) spectrometer (UV1800PC, China). The ICG loading content was ascertained from a standard curve generated with known ICG concentration gradients. The loading efficiency was calculated via the following formula:

$$Loading efficiency\left( \% \right)=\frac{M_{l}}{M_{t}}\times100\%$$

where $M_{l}$ represents the mass of loaded ICG. $M_{t}$ represents the total mass of ICG.

To evaluate the *in vitro* release rate of ICG, 3 mL of ICG@FCS solution was placed in a dialysis bag with a cutoff molecular weight of 3500 kDa, and then the dialysis bag was placed in a 50 mL centrifuge tube containing 10 mL of PBS. The centrifuge tube was then placed in a 37 °C water bath shaker for incubation. At 0, 1, 2, 3, 4, 5, 10, 20, and 30 minutes, 1 mL of PBS was added, and 1 mL of fresh PBS solution was added. Finally, the concentration of ICG released from the ICG@FCS nanoparticles was determined via UV–Vis–NIR at a wavelength of 781 nm. The release in simulated gastric fluid (SGF) followed the same experimental steps as described above in PBS.

***Measurement of ^1^O_2_ Generation:*** We employed a singlet oxygen sensor green (SOSG) assay kit to measure the ^1^O_2_ generation of ICG@FCS *in vitro*. SOSG (2 μM) was dissolved in deoxygenated PBS (pH 7.4) and then introduced into solutions containing free ICG, FCS, and ICG@FCS. Subsequently, the solutions were subjected to US irradiation (1 MHz, 1.5 W/cm^2^) for 0, 1, 5, 10 and 15 minutes under light-shielded conditions. The fluorescence intensity of the solutions was measured via a fluorescence spectrometer at an excitation wavelength of 785 nm.

***Cell*** ***Culture, H. pylori Strains, and Mice:*** HFE-145 cells were cultured in DMEM/F12 medium (Gibco, CA, USA) supplemented with 10% fetal bovine serum (FBS) and 1% penicillin/streptomycin and incubated in a CO_2_ incubator with 5% CO_2_ at 37 °C. *H. pylori*, which had been cryopreserved at -80 °C, was revived on blood agar plates containing 5% sheep blood and a 1% antibiotic mixture composed of 5 μg/mL amphotericin, 10 μg/mL vancomycin, 5 μg/mL polymyxin B sulfate, and 5 μg/mL trimethoprim-treated lactate. The bacteria were then cultured in Brucella broth supplemented with 10% FBS and 0.5% of the aforementioned antibiotic mixture and incubated on a shaker. Male C57BL/6 mice aged five to six weeks and weighing 15 to 25 g were obtained from Jiangsu GemPharmatech LLC (Nanjing, China) and housed at the Pancreatic Disease Research Institute of Changhai Hospital (Shanghai, China) under a specific pathogen-free condition. The house was maintained at 18 to 22 °C and 55% humidity with a 12-hour light/dark cycle.

***Cytotoxicity and Live/Dead Cell Staining Assay:*** HFE-145 cells were plated at a density of 1 × 10^4^ cells per well in a 96-well plate and cultured overnight. Subsequently, the medium was replaced with fresh medium containing varying concentrations of ICG@FCS (150, 200, 250, 300, or 350 μg/mL), and the cells were incubated for 24, 48, or 72 hours. Cell viability was assessed using the Cell Counting Kit-8 (CCK-8) assay, where 100 μL of a 10% CCK-8 solution was added to each well. After a 1-hour incubation at 37 °C, the absorbance at 450 nm was measured by using a microplate reader (Molecular Devices SpectraMax® i3, USA). Cell viability was calculated using the following formula:

$$Cell viability \left( \% \right)=\frac{{OD}_{experimental groups}-{OD}_{blank groups}}{{OD}_{control groups}-{OD}_{blank groups}}\times100\%$$

Similarly, the CCK-8 assay was utilized to evaluate the impact of different exposure times to US treatment on cell toxicity. After coincubation with the material, the cells were subjected to US for durations of 1, 5, 10, or 15 minutes. Following treatment, the cells were further incubated for 24, 48, or 72 hours before proceeding with the CCK-8 assay as previously described.

Additionally, the cells were stained with Calcein AM and PI dyes for the live/dead cell staining assay. After a 30 minutes incubation at 37 °C, the cells were observed under a fluorescence inverted microscope (Leica, DMIL LED, Wetzlar, Germany).

***Hemolysis Evaluation:*** Blood was collected from healthy rats and placed into heparinized anticoagulant tubes. After centrifugation at 3000 rpm for 15 minutes, the lower layer of blood cells was isolated. 2 mL of the collected blood was then diluted with physiological saline to achieve a total volume of 50 mL. Subsequently, 300 μL of the diluted blood cells were transferred to tubes. The positive control group was treated with 1.2 mL of deionized water, while the negative control group received 1.2 mL of physiological saline. The experimental groups were exposed to 1.2 mL of physiological saline containing varying concentrations of ICG@FCS. Following a 2-hour incubation at 37 °C, the absorbance of the supernatant was measured at 540 nm via a microplate reader. The formula for calculating the rate of hemolysis is as follows:

$Hemolysis rate \left( \% \right)=\frac{{OD}_{experimental groups}-{OD}_{negative control group}}{{OD}_{positive control group}-{OD}_{negative control group}}\times100\%$

***Mucosal Permeation Experiment:*** Following previously reported methods,^[3]^ the experiments were conducted via a transwell system. Briefly, freshly slaughtered pig mucosa (25 mg) was laid flat on a polycarbonate filter membrane with a pore size of 3 μm, and a 1 mL suspension of *H. pylori* was added to the receptor chamber. Subsequently, 2 mL each of ICG and ICG@FCS were added to the mucosal surface, and the samples were incubated at 37 °C on a shaker at 100 rpm. For the first set of experimental plates, US treatment was applied for 10 minutes after 2 hours of incubation, after which 200 μL of the sample was removed from the receptor chamber to measure the fluorescence intensity. For the second set of experimental plates, 200 μL of sample was taken every 6 hours to measure the OD_600_ value, which was used to calculate the bacterial concentration. The apparent permeability coefficient (P_app_) was determined via the following formula:

$$P_{app}=\frac{dQ}{dt}\times\frac{1}{A\times C}\times100\%$$

where dQ/dt is the flux of materials from the donor side to the receiver side, A is the membrane area (cm^2^), and C is the initial concentration of materials in the donor chamber.

***In Vitro and in Vivo Targeted Experiments:*** 1 mL (1×10^4^ CFU/mL) *H. pylori* suspensions were cocultured with PBS (1 ml), ICG@CS (without FS) (1 mL, 600 μg/mL), or ICG@FCS (1 mL, 600 μg/mL) for 0, 10, 15, or 20 minutes and then centrifuged at 425 × g for 10 minutes, after which the supernatant was discarded (to remove untargeted materials). The bacterial suspensions were resuspended in PBS, and the fluorescence intensity was measured via a microplate reader at an excitation wavelength of 785 nm and an emission wavelength of 810 nm. Additionally, we used TEM images to observe the morphological changes in *H. pylori* after different treatments, including 10 minutes of US alone; coincubation with ICG@FCS for 0, 10, and 15 minutes; and 15 minutes of incubation followed by 10-minute US. This allowed us to assess the targeted binding ability of ICG@FCS to *H. pylori*.

For *in vivo* targeted evaluation, we first fasted the mice for 1 day. Then, normal (n=3) or *H. pylori*-infected (n=3) mice were orally administered Cy7-labeled ICG@FCS (MCE, HYD0825). After 8 hours, the gastric tissues were imaged via an IVIS imaging system (Tanon ABL X6, China).

***In Vitro Anti-H. pylori Assay:*** A suitable amount of *H. pylori* was collected from a Columbia blood plate using an inoculation loop and resuspended in Brucella broth. The diluted bacterial suspension was transferred to the cuvette of a spectrophotometer set to a wavelength of 600 nm to measure the optical density (OD_600_). The absorbance was proportional to the bacterial concentration, which was controlled at 10^6^ CFU/mL. Subsequently, 1 mL of bacterial suspension was cocultured with 9 mL of Brucella broth, 1 mL of FBS, 50 μL of mixed antibiotics, and 3.3 mg of ICG@FCS. After 1 hour of oscillation incubation in a three-gas incubator, the mixture was centrifuged at 5000 rpm for 3 minutes. The supernatant was discarded, and the bacterial pellet was resuspended in PBS. The suspension was then subjected to US treatment for 1, 5, 10, or 15 minutes. Porcine skin tissue (5 mm thick) was placed between the US probe and the bacterial suspension to simulate an *in vitro* US environment. After treatment, the OD_600_ was measured to calculate the bacterial concentration. Additionally, 20 μL of the diluted bacterial suspension was evenly spread on a blood agar plate and incubated for 72 hours for colony counting and photography.

To assess bacterial surface damage, after the bacteria were cocultured with the material, the mixture was centrifuged at 5000 rpm for 3 minutes. The supernatant was removed, and the bacterial pellet was washed three times with PBS. Following removal of the supernatant, a precooled 2.5% glutaraldehyde solution was slowly added along the tube wall to fix the bacteria at 4 °C for more than 12 hours. The fixed bacteria were subsequently dehydrated in graded alcohol concentrations (30%, 50%, 60%, 70%, 80%, 90%, 95%, 100%) for 10 minutes each, dispersed in tert-butanol, freeze-dried, and coated with gold for scanning electron microscopy observation of bacterial morphology.

Similarly, after the bacterial suspension was cocultured with the sample, SYTO 9/PI Live/Dead Bacterial Double Staining Reagent was added, followed by incubation in low light for 15 minutes. Observation and imaging were conducted under an ortho-fluorescence microscope (MshOt, MF43-N, China).

***Bacterial Biofilm Inhibition:*** *H. pylori* with an OD_600_ value of 0.1 was inoculated into cocultures with 5% Brucella broth medium on confocal culture plates. The cultures were then statically incubated for 3 days in a triple-air incubator, resulting in the formation of a membrane-like structure at the bottom of the dish. Subsequently, the culture medium was replaced with PBS, ICG@FCS, US, or ICG@FCS+US, and the efficacy of biofilm eradication was evaluated using crystal violet staining, confocal laser scanning microscopy (CLSM, Olympus, FV1200), and scanning electron microscopy (SEM, ZEISS Sigma 300, Germany), as previously described.^[4]^

***Construction of the Intracellular H. pylori Model*:** Approximately 4×10^5^ cells were seeded into 6-well plates and incubated overnight. *H. pylori* was added to the cell culture medium at a bacteria-to-cell ratio of 100:1 and cocultured for 6 hours to facilitate bacterial invasion into the cells. The cells were subsequently cocultured with medium containing 100 mg/L gentamicin for 1.5 hours to eliminate extracellular bacteria. The intracellular levels of *H. pylori* were then assessed via both PCR and immunofluorescence methods.

PCR: An intracellular *H. pylori* infection model was constructed as previously described. Then, *H. pylori* DNA within the cells was extracted using the Wizard Genomic DNA Purification Kit (Promega, A1120). The quantity of *H. pylori* within the cells was determined by establishing the ratio of *H. pylori*-specific 16S DNA to GAPDH, following the method described in a previous reference.^[5]^ The primers used to detect *H. pylori* DNA were as follows: forward primer: 5'-TTTT GTT AGA GAA GAT AAT GAC GGT ATC TAA C-3' and reverse primer: 5'-CAT AGG ATT TCA CAC CTG ACT GAC TAT C-3'. The primers for detecting human GAPDH DNA were as follows: forward primer, 5'-GAC TTC AAC AGC GAC ACC C-3'; reverse primer, 5'-AGA AGA TGA AAA GAG TTG TCA GGG C-3'.

Immunofluorescence staining: After modeling as described above, the cells were fixed with 4% paraformaldehyde at room temperature for 30 minutes and washed three times with PBS. The samples were then permeabilized with 0.5% Triton X-100 for 15 minutes and washed three times with PBS. The cells were incubated overnight at 4 °C with 3% BSA and a primary antibody (Abcam, 20459). The next day, the cells were warmed to room temperature for 30 minutes, washed three times with PBS, and incubated with a secondary antibody (Abcam, 150080) at room temperature for 1 hour. After washing with PBS, DAPI and an antifade agent (Thermo Fisher, P36981) were added. The cells were then observed via CLSM.

***Killing*** ***Test of Intracellular H. pylori:*** After the intracellular *H. pylori* model was constructed as described above, the model was cultured for an additional 24 hours following treatment with PBS, rapamycin, ICG@FCS, US, or ICG@FCS+US. The survival of intracellular *H. pylori* was assessed via PCR and immunofluorescence staining.

***Autophagy-related Experiments:*** Western blotting was utilized to evaluate autophagy levels in cells following nanomaterial treatment. After the intracellular *H. pylori* model was constructed and the cells were treated with nanomaterials, proteins were extracted from the cells and detected via a BCA kit. Immunoblotting with primary antibodies against SQSTM1/p62 (Servicebio, GB11531) and LC3-B (CST, 3868S) was used to detect autophagy-related proteins. The methodologies used are detailed in previous literature.^[4b]^

***H. pylori-infected Mouse Model*:** The animal model was constructed according to methods described in previous literature.^[4b]^ Briefly, 300 μL of *H. pylori* suspension (10^8^ CFU/mL) was administered to each mouse via gavage every other day for two weeks. After the gavage period, the mice were maintained under standard housing conditions for an additional four weeks. Five mice were subsequently randomly selected for validation. The excised gastric tissue was homogenized in physiological saline and diluted, and the resulting suspension was plated onto selective plates. The plates were incubated at 37 °C in a 5% CO₂ environment for 48 hours. After incubation, the colonies on the plates were examined. The bacterial suspension was then inoculated into a rapid urease test reagent to observe any color changes. Additionally, gastric tissue was subjected to H&E staining to assess the morphology of *H. pylori*.

***Biological Safety Test*:** After the mice underwent different treatments, their hearts, livers, spleens, lungs, and kidneys were collected for H&E staining. This part of the experiment was conducted by Wuhan Servicebio Technology Co., Ltd. (China). Additionally, serum was collected for liver and kidney function tests, which were carried out by Daixuan Biotechnology Co. (Shanghai, China).

***In Vivo Anti-H. pylori Assay:*** *H. pylori*-infected mice were randomly divided into 5 groups (n=6): the PBS, ICG@FCS, US, antibiotic (clarithromycin, 14.3 mg/kg/day), and ICG@FCS+US groups. The US and ICG@FCS+US groups were exposed to US for 10 minutes. Treatment was administered once every other day for two weeks. On the second day after treatment, all the mice were euthanized, and their stomachs were subjected to plate coating and H&E staining to assess the *in vivo* antibacterial effect.

***Cell Scratch Assay:*** During the exponential growth phase, HFE-145 cells were treated with trypsin for digestion. Then, the 6-well plates were washed with PBS, and 2 mL of culture medium was added to each well. The digested cells were evenly distributed into 6-well plates, with an additional 1 mL of cell-containing culture medium added to each well. The plates were returned to the 37 °C incubator for continued cultivation, with daily monitoring of cell growth. Once the cells completely covered the wells, a scratch was made on the monolayer using the tip of a 10 µL pipette. The cells were then gently washed three times with PBS to remove any detached cells. Next, the cells were cocultured with ICG@FCS (300 µg/mL) at 37 °C, and changes in the growth of the scratch area were observed at 0, 24, and 48 hours.

***Mucosal Repair Experiment in Vivo:*** Gastric tissues from the normal control, *H. pylori* infection, ICG@FCS, US, antibiotic, and ICG@FCS+US groups of mice were subjected to H&E staining and fluorescence staining to assess the *in vivo* repair of gastritis. The H&E staining procedure was the same as above. Additionally, gastric pathology was scored for gastritis using the Sydney System.^[6]^ Fluorescence staining was conducted by Wuhan Servicebio Technology Co., Ltd. (China). The apoptosis of gastric epithelial cells was evaluated via β-catenin and Ki67 staining, whereas gastric mucosal repair was assessed via Occludin and Claudin staining.

For RNA extraction from some gastric tissues, the following protocol was used to perform qPCR to detect the relative expression levels of Claudin, ZO-1, β-catenin, Ki-67, JAM-1, and Occludin. Tissue RNA was extracted according to the TRIzol™ protocol and the manufacturer’s instructions. Approximately 60 mg of tissue was carefully weighed and ground into a fine powder via liquid nitrogen in a homogenizer for 5 minutes. After a brief 5-minute incubation, the homogenate was centrifuged at 12,000 × g for 5 minutes at 4 °C. The resulting supernatant was carefully transferred to a fresh tube containing 0.3 mL of chloroform/isoamyl alcohol (24:1). Following a subsequent 10-minute centrifugation (4 °C, 12,000 rpm), the supernatant was carefully transferred to a new tube containing an equal volume of isopropanol and centrifuged again for 20 minutes (4 °C, 13,600 rpm). The resulting RNA pellets were washed twice with 1 mL of 75% ethanol, with residual ethanol removed by brief centrifugation for 2 minutes (4 °C, 13,600 rpm), followed by a 5-minute air-drying step in a biosafety cabinet. Finally, the RNA was dissolved in 25–100 μL of diethyl pyrocarbonate-treated water. The RNA sample quality and quantity were then determined via an Agilent 2100 Bioanalyzer (Thermo Fisher Scientific, MA, USA).

***Gut Microbiota Analysis*:** Fecal samples were collected from the mice in each experimental group for analysis of gut microbiota abundance and diversity via 16S rRNA sequencing. This analysis was performed by Daixuan Biotechnology Co. (Shanghai, China). Briefly, genomic DNA was extracted from samples via the MagPure Soil DNA LQ Kit (Magan) according to the manufacturer's instructions. The concentration and purity of the extracted DNA were assessed via a NanoDrop 2000 (Thermo Fisher Scientific, USA) and agarose gel electrophoresis, and the resulting DNA was stored at -20 °C. For bacterial 16S rRNA gene amplification, the extracted genomic DNA was used as a template, with specific primers containing barcodes and Takara Ex Taq high-fidelity enzymes. The universal primers 343F (5'-TACGGRAGGCAGCAG-3') and 798R (5'-AGGGTATCTAATCCT-3') were used to amplify the V3-V4 variable regions of the 16S rRNA gene for bacterial diversity analysis. The amplicon quality was visualized using gel electrophoresis, and PCR products were purified using Agencourt AMPure XP beads (Beckman Coulter Co., California, USA) and quantified with the Qubit dsDNA assay kit (Thermo Fisher Scientific, Massachusetts, USA). Sequencing libraries were generated using NEBNext® Ultra^TM^ II FS DNA Library Pre Kit (NEB #E6177, New England Biolabs, Massachusetts, USA) for Illumina, following manufacturer’s recommendations and index codes were added. The library quality was assessed on the Qubit^@^ 2.0 Fluorometer and Agilent Bioanalyzer 2100 system. Then, sequencing was performed on an Illumina NovaSeq6000 with paired-end 250 bp read cycles.

After sequencing, Cutadapt software was used to trim primer sequences from the raw data. The DADA2 plugin, which uses QIIME 2 (2020.11),^[7]^ was then applied for quality filtering, denoising, merging, and chimera removal to produce representative sequences and an amplicon sequence variant (ASV) abundance table. The QIIME 2 software package was used to select representative sequences, which were annotated by alignment with the Silva (version 138) database. Taxonomic annotation was performed via the q2-feature-classifier plugin with default parameters. Both α and β diversity analyses were conducted via QIIME 2, and LEfSe was used for differential abundance analysis of the species. The raw data will be made available upon reasonable request to the corresponding author.

***Transcriptomic Analysis*:** Gastric tissues from the mice treated with PBS or ICG@FCS+US were obtained for transcriptional analysis to identify genetic differences in the mice treated with ICG@FCS+US. The experiments were conducted by Major Biomedical Technology Co. (Shanghai, China).

***Statistical Analysis*:** All the data are expressed as the means ± standard deviations. Each experiment included a sample size of n ≥ 3 for statistical analysis. Differences between two groups were compared via Student's t test, whereas differences among three or more groups were compared via one-way ANOVA. Statistical significance was defined as *P* < 0.05 (ns: not significant, * *P* < 0.05, ** *P* < 0.01, and *** *P* < 0.001). All statistical analyses were conducted using R statistical software 3.6.1 ([www.r-project.org](http://www.r-project.org)).

**References**

[1] Y.-H. Lin, S.-C. Tsai, C.-H. Lai, C.-H. Lee, Z. S. He, G.-C. Tseng, *Biomaterials* **2013**, *34* (18), 4466.

[2] Z. Chen, X. Zheng, J. Zhao, J. Tang, L. Hu, S. Wang, *International Journal of Pharmaceutics* **2023**, *636*, 122848.

[3] Y. Shen, Y. Zou, X. Chen, P. Li, Y. Rao, X. Yang, Y. Sun, H. Hu, *Journal of Controlled Release : Official Journal of the Controlled Release Society* **2020**, *328*, 575.

[4] a) J. Yu, Z. Guo, J. Yan, C. Bu, C. Peng, C. Li, R. Mao, J. Zhang, Z. Wang, S. Chen, M. Yao, Z. Xie, C. Yang, Y. Y. Yang, P. Yuan, X. Ding, *Adv Sci (Weinh)* **2023**, *10* (20), e2206957; b) Y. Lai, T. Zhang, X. Yin, C. Zhu, Y. Du, Z. Li, J. Gao, *Acta Pharmaceutica Sinica B* **2024**.

[5] W. Hu, L. Zhang, M. X. Li, J. Shen, X. D. Liu, Z. G. Xiao, D. L. Wu, I. H. T. Ho, J. C. Y. Wu, C. K. Y. Cheung, Y. C. Zhang, A. H. Y. Lau, H. Ashktorab, D. T. Smoot, E. F. Fang, M. T. V. Chan, T. Gin, W. Gong, W. K. K. Wu, C. H. Cho, *Autophagy* **2019**, *15* (4), 707.

[6] A. Andrew, J. I. Wyatt, M. F. Dixon, *Histopathology* **1994**, *25* (4), 317.

[7] E. Bolyen, J. R. Rideout, M. R. Dillon, N. A. Bokulich, C. C. Abnet, G. A. Al-Ghalith, H. Alexander, E. J. Alm, M. Arumugam, F. Asnicar, Y. Bai, J. E. Bisanz, K. Bittinger, A. Brejnrod, C. J. Brislawn, C. T. Brown, B. J. Callahan, A. M. Caraballo-Rodríguez, J. Chase, E. K. Cope, R. Da Silva, C. Diener, P. C. Dorrestein, G. M. Douglas, D. M. Durall, C. Duvallet, C. F. Edwardson, M. Ernst, M. Estaki, J. Fouquier, J. M. Gauglitz, S. M. Gibbons, D. L. Gibson, A. Gonzalez, K. Gorlick, J. Guo, B. Hillmann, S. Holmes, H. Holste, C. Huttenhower, G. A. Huttley, S. Janssen, A. K. Jarmusch, L. Jiang, B. D. Kaehler, K. B. Kang, C. R. Keefe, P. Keim, S. T. Kelley, D. Knights, I. Koester, T. Kosciolek, J. Kreps, M. G. I. Langille, J. Lee, R. Ley, Y.-X. Liu, E. Loftfield, C. Lozupone, M. Maher, C. Marotz, B. D. Martin, D. McDonald, L. J. McIver, A. V. Melnik, J. L. Metcalf, S. C. Morgan, J. T. Morton, A. T. Naimey, J. A. Navas-Molina, L. F. Nothias, S. B. Orchanian, T. Pearson, S. L. Peoples, D. Petras, M. L. Preuss, E. Pruesse, L. B. Rasmussen, A. Rivers, M. S. Robeson, P. Rosenthal, N. Segata, M. Shaffer, A. Shiffer, R. Sinha, S. J. Song, J. R. Spear, A. D. Swafford, L. R. Thompson, P. J. Torres, P. Trinh, A. Tripathi, P. J. Turnbaugh, S. Ul-Hasan, J. J. J. van der Hooft, F. Vargas, Y. Vázquez-Baeza, E. Vogtmann, M. von Hippel, W. Walters, Y. Wan, M. Wang, J. Warren, K. C. Weber, C. H. D. Williamson, A. D. Willis, Z. Z. Xu, J. R. Zaneveld, Y. Zhang, Q. Zhu, R. Knight, J. G. Caporaso, *Nat Biotechnol* **2019**, *37* (8), 852.

Figure S1. The particle size distribution of FCS.

Figure S2. The standard curve of ICG.

Figure S3. Live/dead cell staining of HFE-145 cells after coculture with different concentrations of ICG@FCS for 3 days; bars represent 100 µm.

Figure S4. Cell viability was assessed after co-culturing with ICG@FCS, followed by US treatment at different time intervals for 1, 2, and 3 days.

Figure S5. The anti-*H. pylori* activity *in vitro* transwell model (n = 3).


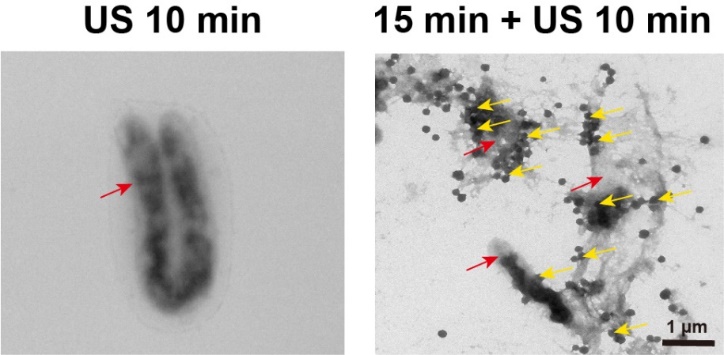


Figure S6. TEM images of US 10 minutes (left), and US 10 minutes after ICG@FCS co-incubated with *H. pylori* for 15 minutes (right) (scale bar: 1 µm).

Figure S7. Quantitative analysis of *H. pylori* counts on agar plates. The data are presented as the means ± SDs (n = 3), **P <* 0.05, ***P <* 0.01, ****P <* 0.001. ns, not significant.


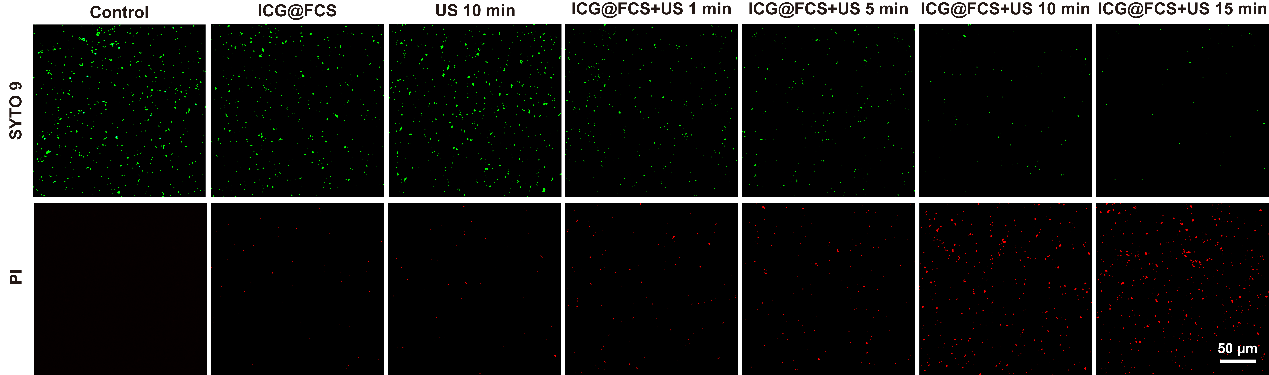


Figure S8. Live (green fluorescence)/dead (red fluorescence) bacterial staining of *H. pylori*; bars represent 50 μm.

Figure S9. Validation of the *H. pylori-*infected mouse model. (a) Blood agar plate coating image; the bars represent 1 cm. (b) Urease test; bars represent 5 mm. (c) H&E staining; bars represent 100 µm.


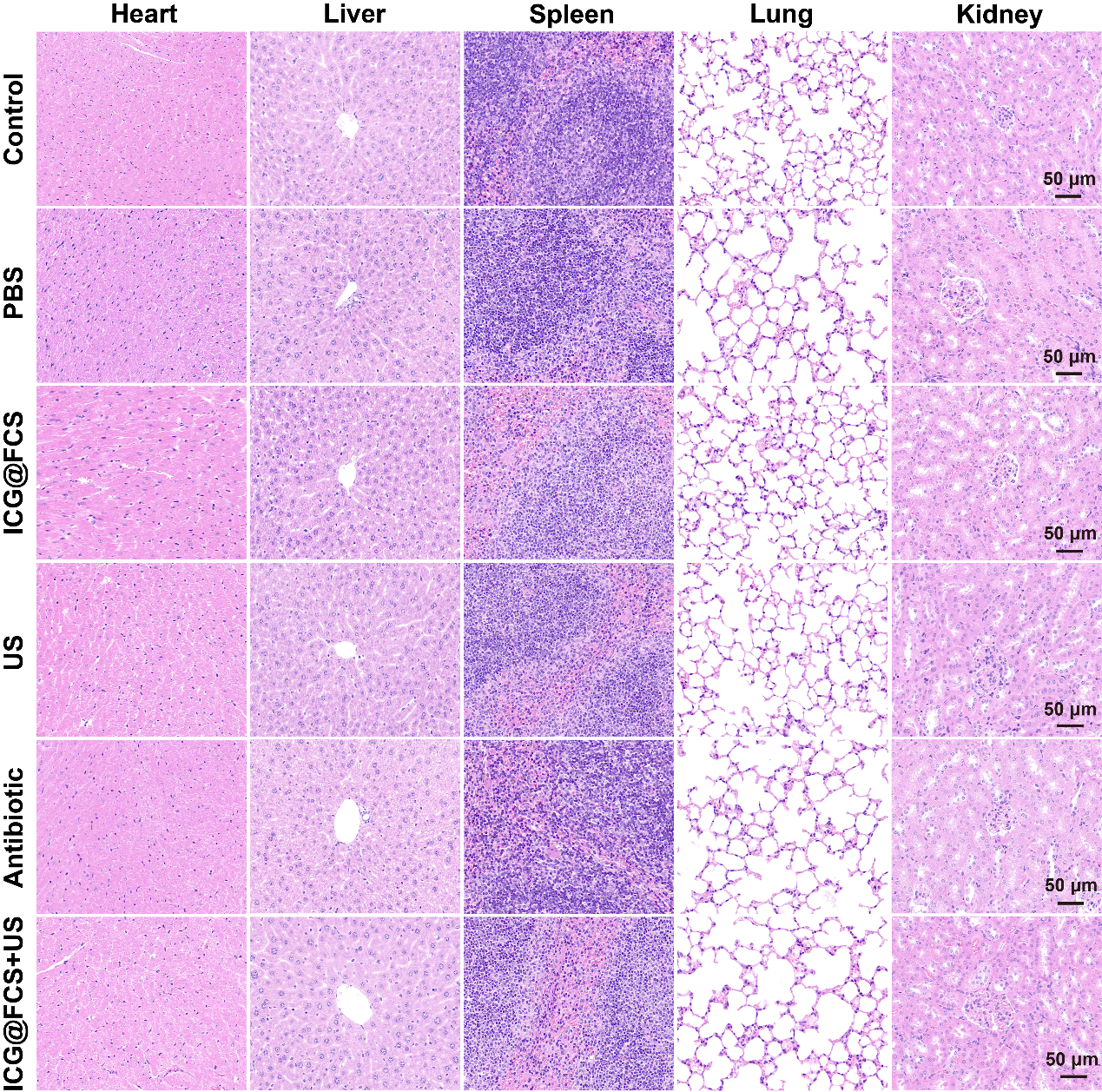


Figure S10. H&E staining of the heart, liver, spleen, lung, and kidney of mice after treatment with PBS, ICG@FCS, US, antibiotics (clarithromycin, 7.5 mg/kg/day), or ICG@FCS+US for two weeks; bars represent 50 µm.


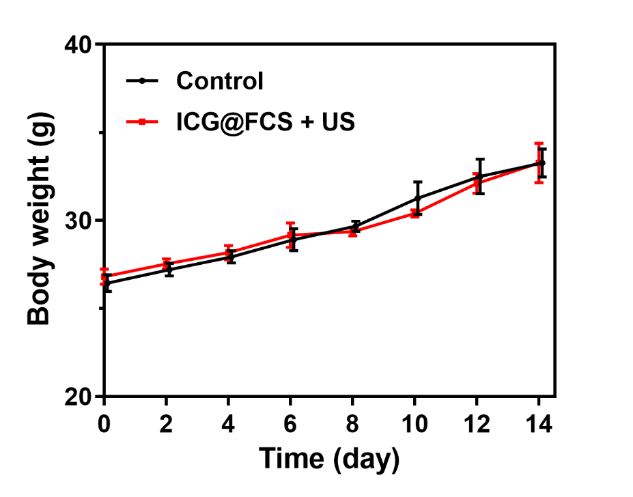


Figure S11. Body weights of the mice after treatment with PBS or ICG@FCS+US for two weeks (n = 3).


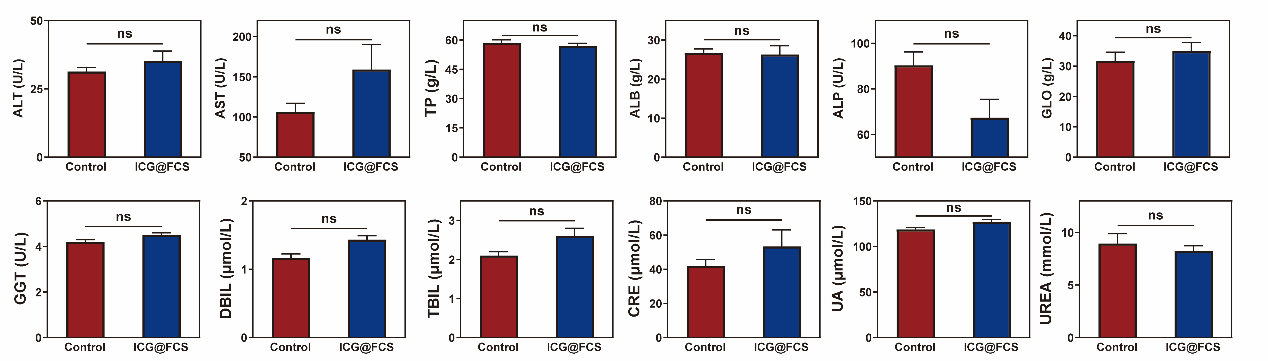


Figure S12. Liver and kidney function assays in mice after ICG@FCS treatment. The data are presented as the means ± SDs (n = 3). ns, not significant.


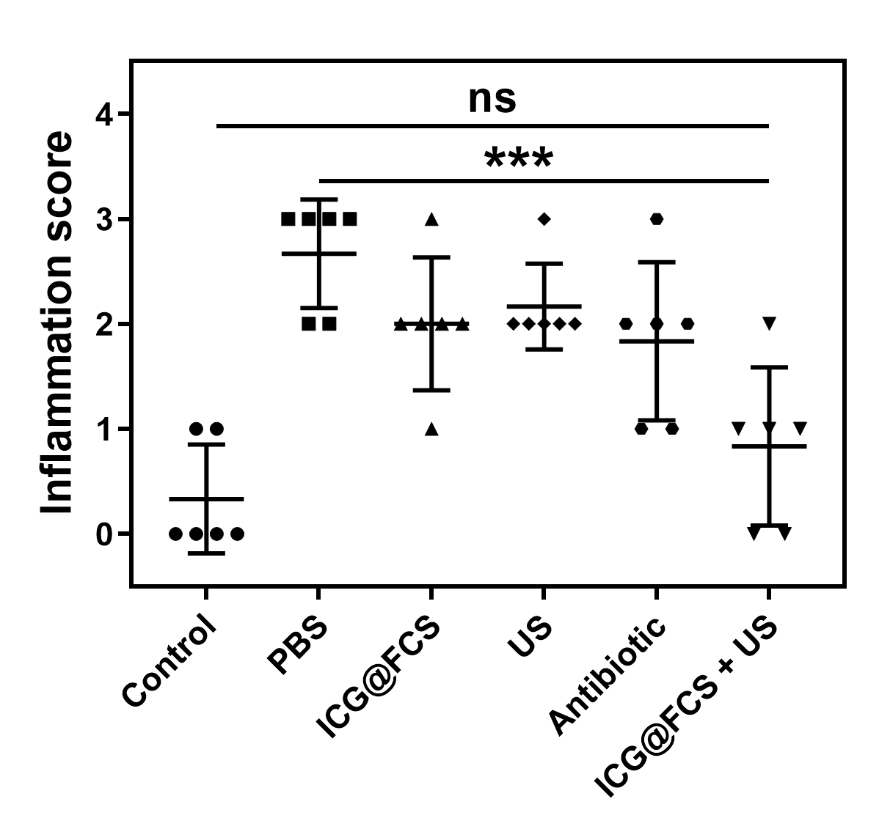


Figure S13. Inflammation scores were obtained by analyzing H&E-stained images, as shown in Figure 6C. The data are presented as the means ± SDs (n = 6), **P <* 0.05, ***P <* 0.01, ****P <* 0.001. ns, not significant


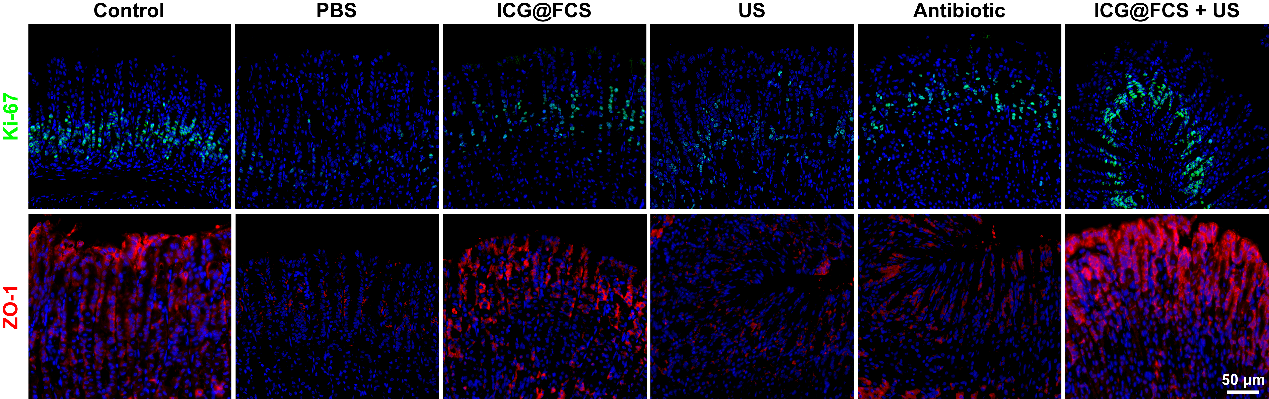


Figure S14. Epithelial repair of the mouse gastric mucosa was observed via ZO-1 fluorescence staining. Fluorescence staining of Ki-67 was performed to detect apoptosis of gastric epithelial cells; scale: 50 μm.


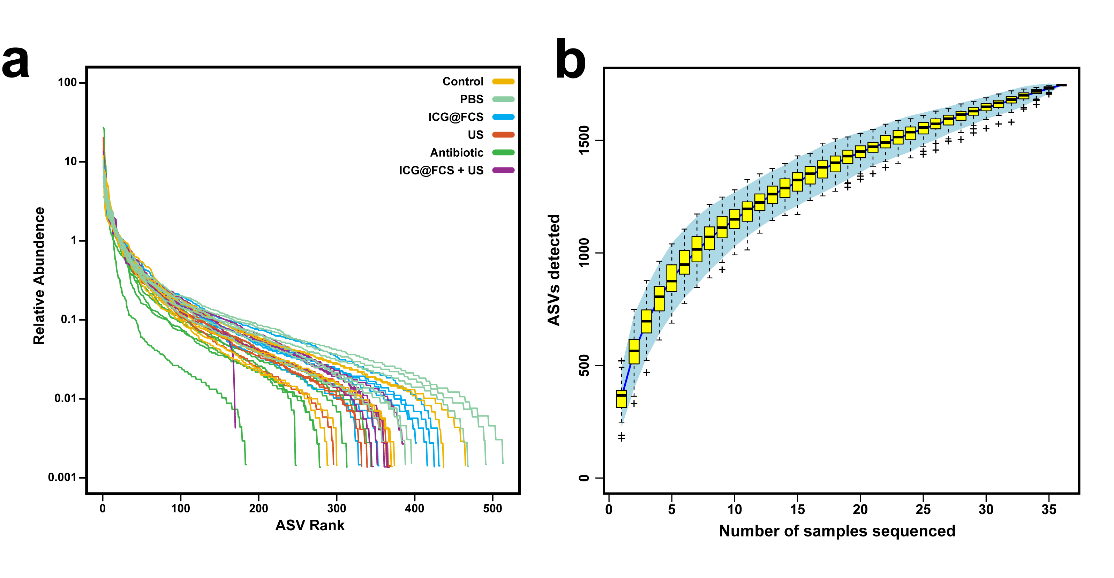


Figure S15. Rank-abundance analysis (a) and Specaccum species accumulation curve (b) of mouse fecal samples to assess the adequacy of sampling size and evaluate species richness (n=6).


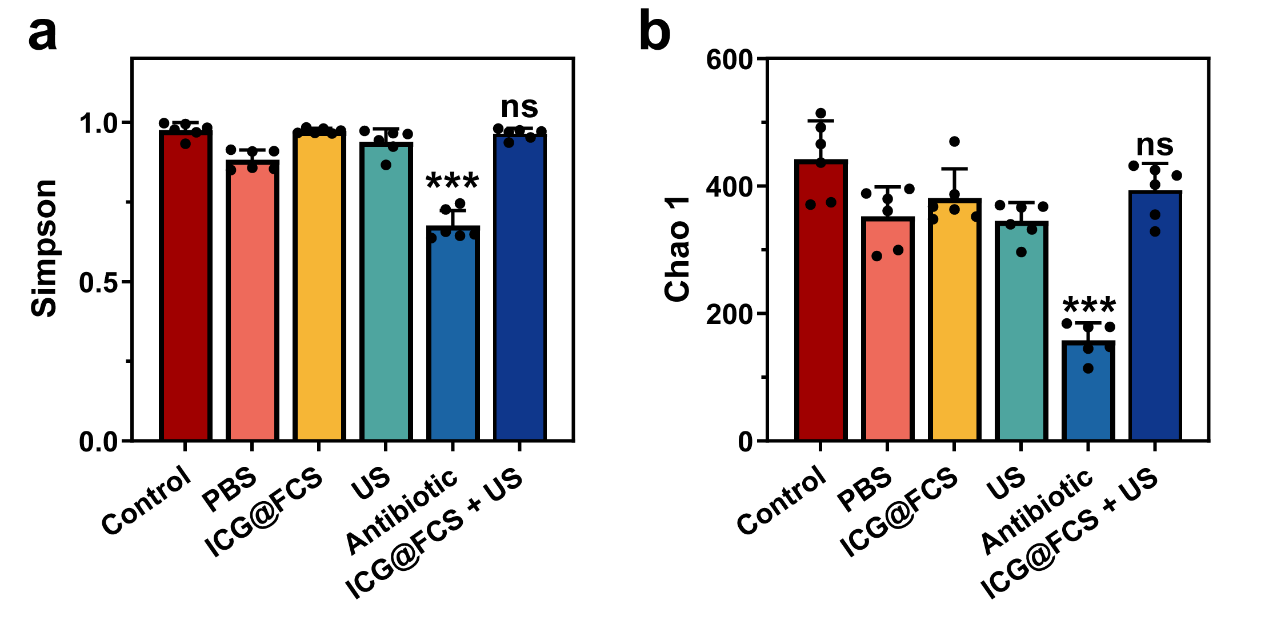


Figure S16. Determination of the Simpson index and Chao 1 index in mouse feces to analyze microbial richness. The data are presented as the means ± SDs (n = 6), **P <* 0.05, ***P <* 0.01, ****P <* 0.001. ns, not significant.


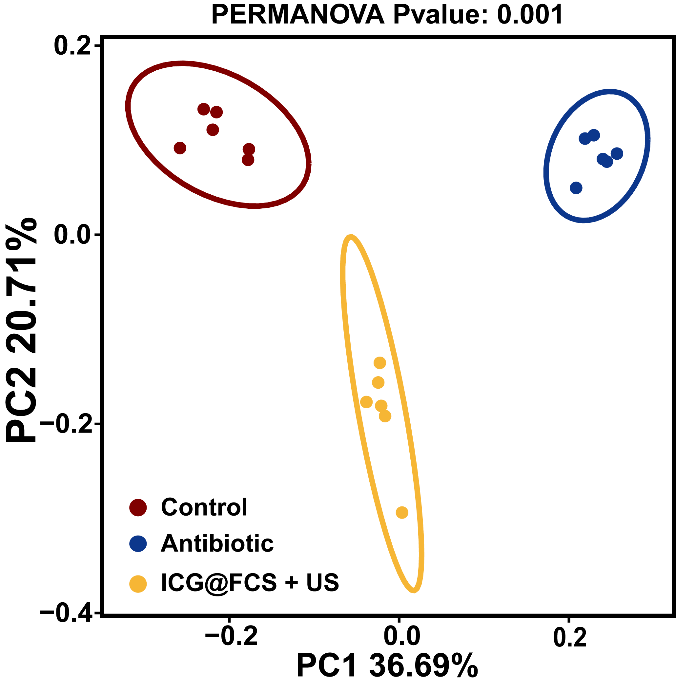


Figure S17. PCoA analysis of intestinal microbiota β diversity after antibiotic and ICG@FCS+US treatments (n = 6).

| **Table S1. Primer sequence (5′- 3′) for the qPCR.** | |
| --- | --- |
| **Primer name** | **Primer sequence** |
| Mki67 | GCACACTGGCAGAAACATAG |
| Ctnnb1 | GATACTGACCTGTAAATCGTCC |
| Tjp1 | TAAAGCTGTCCCTGTGAGTCCT |
| Ocln | CAGCCCTCAGGTGACTGTTAT |
| F11r | CGAGGCTCTCATAACTACGCT |
| Cldn1 | TGACATTGGTAGACCTGGATT |
